# Multi-isotope evidence of population aggregation in the Natufian and scant migration during the early Neolithic of the Southern Levant

**DOI:** 10.1038/s41598-021-90795-2

**Published:** 2021-06-04

**Authors:** Jonathan Santana, Andrew Millard, Juan J. Ibáñez-Estevez, Fanny Bocquentin, Geoffrey Nowell, Joanne Peterkin, Colin Macpherson, Juan Muñiz, Marie Anton, Mohammad Alrousan, Zeidan Kafafi

**Affiliations:** 1 Department of Archaeology, Durham University, Durham, UK; 2 G.I. Tarha, Departamento de Ciencias Históricas, Universidad de Las Palmas de Gran Canaria, Las Palmas, Spain; 3 Consejo Superior de Investigaciones Científicas, Institución Milá y Fontanals, Barcelona, Spain; 4 Cogitamus Laboratory and CNRS, UMR 7041, ArScAn, Equipe Ethnologie Préhistorique, MSH Mondes, Nanterre, France; 5 Department of Earth Science, Durham University, Durham, UK; 6 Pontificia Facultad de San Esteban de Salamanca, Salamanca, Spain; 7 Université Paris 1, Panthéon-Sorbonne, Paris, France; 8 CNRS, UMR 7206, Musée de l’Homme, Éco-Anthropologie et Ethnologie, Paris, France; 9 Department of Anthropology, Yarmouk University, Irbid, Jordan; 10 Department of Archaeology, Yarmouk University, Irbid, Jordan

**Keywords:** Psychology and behaviour, Socioeconomic scenarios, Climate-change adaptation

## Abstract

Human mobility and migration are thought to have played essential roles in the consolidation and expansion of sedentary villages, long-distance exchanges and transmission of ideas and practices during the Neolithic transition of the Near East. Few isotopic studies of human remains dating to this early complex transition offer direct evidence of mobility and migration. The aim of this study is to identify first-generation non-local individuals from Natufian to Pre-Pottery Neolithic C periods to explore the scope of human mobility and migration during the Neolithic transition in the Southern Levant, an area that is central to this historical process. The study adopted a multi-approach resorting to strontium (^87^Sr/^86^Sr), oxygen (δ18O_VSMOW_) and carbon (δ^13^C) isotope ratio analyses of tooth enamel of 67 human individuals from five sites in Jordan, Syria, and Israel. The isotope ratios point both to a significant level of human migration and/or mobility in the Final Natufian which is compatible with early sedentarism and seasonal mobility and with population aggregation in early sedentary hamlets. The current findings, in turn, offer evidence that most individuals dating to the Pre-Pottery Neolithic were local to their respective settlements despite certain evidence of non-locals. Interestingly, isotopic data suggest that two possible non-local individuals benefitted from particular burial practices. The results underscore a decrease in human mobility and migration as farming became increasingly dominant among the subsistence strategies throughout the Neolithic transition of the Southern Levant.

## Introduction

**Figure 1:**
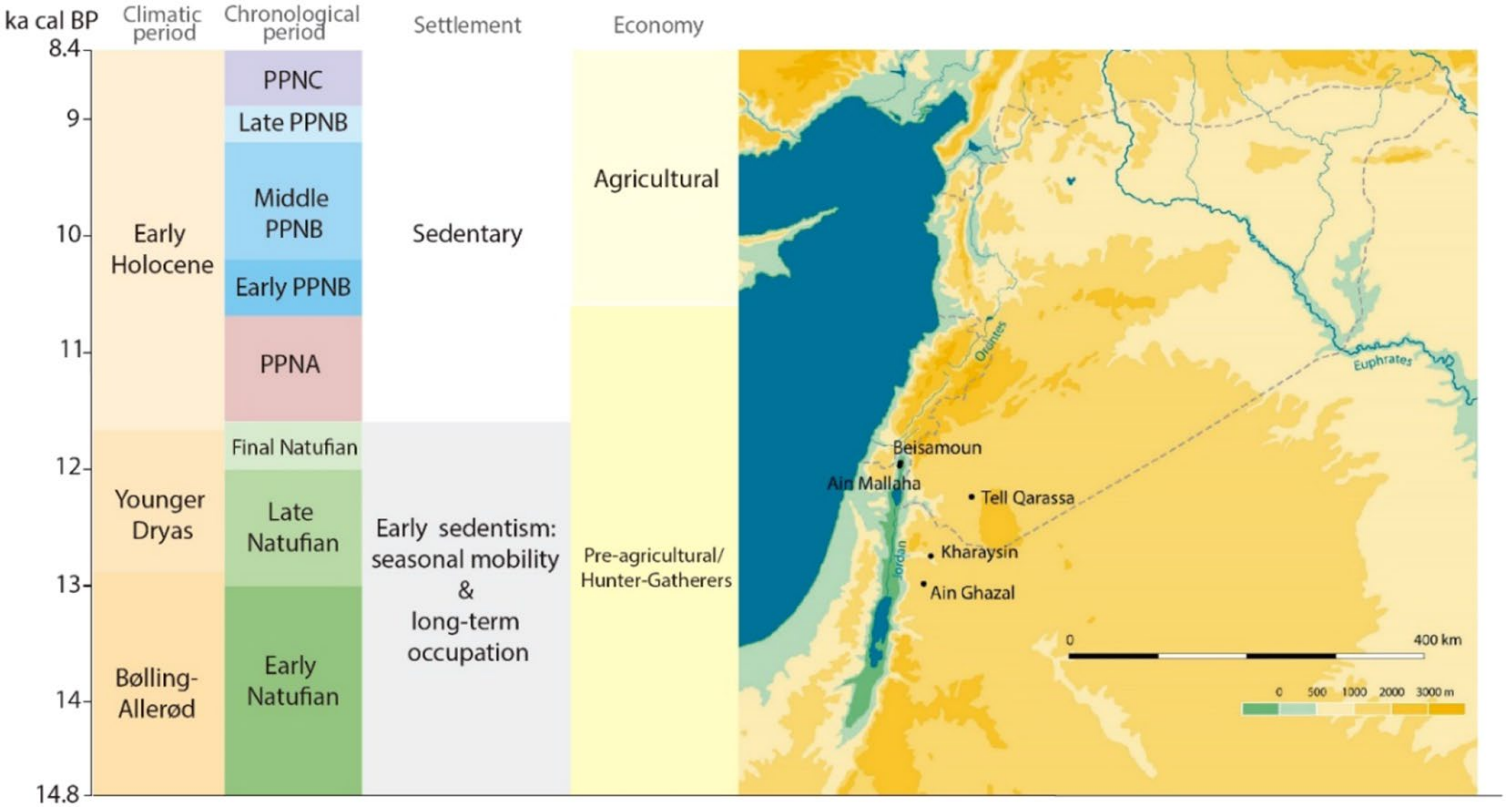
Left: correlation of the chronology (calibrated Before Present), climatic period, chronological period (culture), nature of settlement and economy during the Neolithic transition in the Southern Levant. *PPN* Pre-Pottery Neolithic. Right: Map locating the archaeological sites yielding human enamel samples serving for the analysis. We acknowledge Luis C. Teira for his Near East map (on the right). The figure was generated using Adobe Illustrate CC 2019 (https://www.adobe.com/cn/products/illustrator.html).

The emergence of the Neolithic in the Near East was accompanied by economic, demographic, social and ideological changes which culminated in the development of new ways of life marked by a progressive intensification of food production^1–5^. The Southern Levant was central to this historical process as it offers early evidence of sedentarism and an intensification of the exploitation and control of wild plants and animals^6–8^. The main characteristic of this region is the change from sedentary or semi-sedentary settlements represented by hamlets in the Natufian Period to later extensive mega-sites that emerged in the Middle/Late Pre-Pottery Neolithic B^9,10^ (Fig. 1). This later timeframe saw a consolidation of sedentarism characterised by vast permanent villages with populations in the hundreds^11–13^. Human migration and population aggregation could have played an important role in the development of these sedentary villages^13,14^. A hypothesis suggests that the emergence of Middle/Late Pre-Pottery Neolithic mega-sites in the Jordanian Highlands stemmed from migrations from the Mediterranean heartlands to the western edge of the Jordanian Rift Valley^15^. The hypothesis is based on the notion that population pressure and ecological deterioration promoted migrations. Moreover, certain authors have advanced that the sedentarisation process also favoured substantial demographic growth yielding larger settlements^16^. This significant shift, represented through the Neolithic Demographic Transition model (NDT)^17^, is founded on a relative increase of the proportion of non-adult burials and the totals of the values of female fertility rate (evidenced by the reduction of the birth interval) and a rise in mortality rates. According to those paleodemographers, this escalation is especially noticeable among infants, possible due to new health problems associated with sedentary life (infectious diseases) along with shorter periods of breastfeeding which likewise could have led to an upsurge of maternal fertility^16,17^. The causes underlying this phenomenon remain nonetheless poorly understood^18,19^.

This study also encompasses a critical shift marked by a decrease of mobile hunter-gatherer communities of the Natufian period (c. 15,000–11,500 years BP) in favour of permanent sedentary agrarian settlements dating to the Pre-Pottery Neolithic period (c. 10,300–8000 years BP). The pattern of Natufian human mobility is perceived as a pre-agricultural transition to sedentism with fluctuations in mobility over time^12,20–23^. Nevertheless, specialists do not agree on whether the Natufian period consisted of sedentary or semi-sedentary communities given the highly ambiguous nature of the archaeological evidence and how to really characterise sedentism^24–28^. Interestingly, a strontium analysis of Natufian human remains suggests a model of intensive regionalism and local procurement of food resources, which precludes sedentism^29^. Conversely, a more sedentary lifestyle is detected from Pre-pottery Neolithic A onwards, with extensive evidence of permanent sedentary settlements and domesticates in the Pre-Pottery Neolithic B and C periods^12^.

Short and long-distance exchange networks progressively intensified and gained in complexity from the Epipaleolithic to the mid-9th millennium BP^30^. These networks included mechanisms of social interaction yielding regional patterns that paved the way for the transmission of innovations throughout the Near East^8,31^. This reinforces the notion of interaction spheres and multi-center Neolithization processes during the Neolithic transition^3,6^. Human mobility and migration may have likewise laid the groundwork for complex exchange networks facilitating a rapid transmission of goods, ideas and genes^3^. The migrations to Cyprus from the 11th millennium BP^32^ are an example of this in spite of the fact that the interaction between the island and the mainland ultimately waned in the 8th millennium BP. Genetic exchange is likewise observed in the mitochondrial DNA of individuals from Northern and Southern Levant^33^. The findings of aDNA analyses suggest, nonetheless, that this exchange remained limited^34–37^ as Epigenetic traits from Natufian populations indicate a relative genetic isolation^38,39^. A similar pattern bolstering the notion of limited genetic exchange is also identified at the PPNB settlement of Basta^40^. Epigenetic traits gleaned from human teeth from the PPNB settlement of Kfar HaHoresh, in turn, are interpreted as resulting from biological relationships among females and non-adults but not among adult males, insinuating matrilocal residence patterns and male migration^41^. However, certain scholars highlight the difficulty of offering proof identifying biological kin through dental evidence^42^. It therefore remains unclear if the networks of exchange were occasional, frequent or seasonal, and if they involved the movement of individuals, task groups or whole communities.

There is a gap among the data from the Natufian Period to the PPNC which hinders delving into whether human mobility and migration played substantial roles in the development of sedentary patterns, long-distance exchange and transmission of ideas and practices. The multi-isotope approach of this study thus explores the part played by these factors during the Neolithic transition in the Southern Levant. This meant identifying the strontium (^87^Sr/^86^Sr), oxygen (δ^18^O) and carbon (δ^13^C) isotope ratios in the enamel of 67 human teeth from five sites ranging from the Natufian to PPNC periods. ^87^Sr/^86^Sr ratios in human teeth depend on the geological composition of the area and its influence on the foodstuffs consumed during enamel formation. Depending on tooth type, this takes place among humans anytime from late gestation to early adulthood^43,44^. The human body in fact yields the δ^18^O values of the water resources that vary according to temperature, elevation, food processing and distance to the sea^45,46^. Carbon isotope analyses (δ^13^C) of tooth enamel thus offer evidence of total diet during childhood as their values differ from area to area due to variations among the animals and plants that were consumed^47,48^.

This multi-isotope strategy of this study targeted first-generation non-local individuals from the Natufian to the Pre-Pottery Neolithic C, a timeframe encompassing the Neolithic transition in the Near East (Fig. 1). Specifically, an individual can be considered local when its strontium isotope value falls within the local strontium baseline range, and non-local when the value falls beyond^49^. The local baseline in this study is defined by the biologically available ^87^Sr/^86^Sr signatures and statistical analyses of the human dataset^50^. Local Sr signatures were determined for each site so as to identify specific local/non-local dichotomies as geological complexity differs from one area of the Southern Levant to another (i.e., Jordan Rift Valley vs Jordanian Highlands, Supplementary Material Text S2). Comparing individual values with locally bioavailable strontium and human datasets allows identifying non-local, migrant individuals at a particular site^43,44^. However, this approach could lead to an underestimation of the number of non-local individuals as geological features are very homogenous in certain areas of the Southern Levant which lead to only slight variations of strontium isotopes readings (i.e., Jordanian Highlands, Supplementary Material Text S2)^51^. Conversely, this approach might also overestimate potential non-local individuals at sites located adjacent to the boundaries between geological provinces with distinct ^87^Sr/^86^Sr signatures (i.e., Jordan Rift Valley, Supplementary Material Text S2). Oxygen and carbon isotope ratios are also used to underpin results obtained from strontium ratios.

This approach offers direct evidence of the migration of individuals that cannot be identified by other methods such as archaeogenetics and demographic modelling^52–54^. Furthermore, bone collagen is poorly preserved in individuals from prehistoric sites in the Near East and ancient DNA extractions are rarely successful^36^. In fact, out attempts to collect it for this analysis were unsuccessful. This study avoids this drawback and offers a distinct perspective as to the role of human mobility and migration during the Neolithic transition in the Near East.

## Results

The values of human enamel isotopes ratios are listed in Dataset S1 (Supplementary Material, Table S1). The 67 ratios range from ^87^Sr/^86^Sr 0.70751 to 0.70821 with a mean of 0.70785 ± 0.00001 (2σ) allowing to identify the statistical differences between the different archeological sites (Kruskal–Wallis: H = 53.705, df = 4, *p* < 0.001). The δ^18^O_carb(VSMOW)_ analyses among the 67 individuals reveal a mean of 26.22‰ with a range of 24.38‰ to 32.46‰ (8.08‰ total range). One individual nonetheless displays an outlying value inconsistent with the region (QR09-EF18-V67: δ^18^O_carb(VSMOW)_ = 32.46‰). The isotope ratios, following Chenery et al.^55^, were also converted to drinking water values yielding a δ^18^O_dw(VSMOW)_ range between − 9.86 and 2.98‰ (mean − 6.83‰). Excluding the outlier, the δ^18^O_dw(VSMOW)_ yielded a mean of − 7‰ ranging from − 6.14 to − 3.85‰. These values line up with those of modern precipitation in the Southern Levant and are consistent with individuals who during the formation of their enamel in the course of their childhood resided in the area and consumed local water^56,57^.

**Figure 2:**
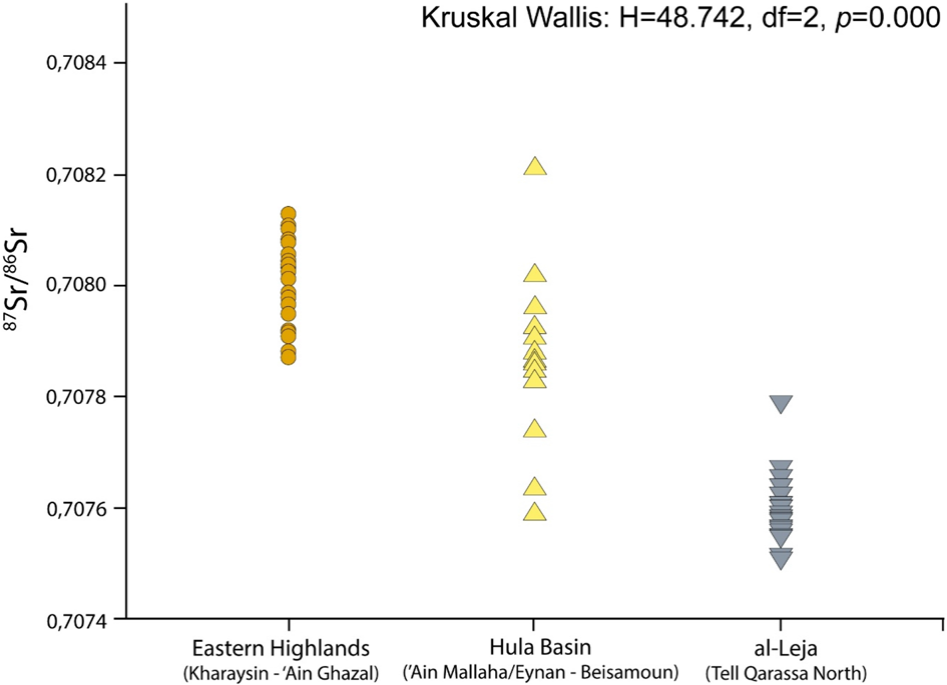
Scatter plot of the ^87^Sr/^86^Sr ratios according to the geological areas identified in the study. The Eastern Highlands includes ^87^Sr/^86^Sr ratios of human samples from the sites of Kharaysin and ‘Ain Ghazal (Cretaceous limestone). The Hula Basin in the upper Jordan Rift Valley corresponds to the sites of Ain Mallaha/Eynan and Beisamoun (Hula Basin: basalt soil, *terra rossa* and Cretaceous limestone) Those from Al-Leja are from the site of Tell Qarassa North (Late Miocene, Pliocene, and Quaternary basalts).

The individuals sampled from the three main geological zones yielded the following average ratios: ^87^Sr/^86^Sr of 0.70762 ± 0.00008 from the volcanic area (Tell Qarassa North), 0.70786 ± 0.00009 from the Hula Basin of the Upper Jordan Rift Valley (‘Ain Mallaha/Eynan and Beisamoun), and 0.70801 ± 0.00001 from the Cretaceous limestone area of the Eastern Highlands (Kharaysin and ‘Ain Ghazal) (Figs. 2, S1). These values underscore statistical differences between the three geological areas (Kruskal Wallis: H = 48.742, df = 2, *p* < 0.001). While the widest distribution corresponds to the Jordan Valley, the volcanic and Eastern Highlands reveal the narrowest distribution. The analysis of the values by site indicates that the wide distribution of samples from the Hula Basin mainly stems from data from ‘Ain Mallaha/Eynan (Fig. 2). The higher radiogenic values are among the samples of the Cretaceous limestone areas of the Jordanian highlands while the lower radiogenic values are from the volcanic district of Tell Qarassa North (Fig. 2, Table S1). It is compelling that strontium isotope ratios from ‘Ain Ghazal are slightly less radiogenic than those of Kharaysin suggesting that potential different local isotopic signatures can be detected in the Cretaceous limestone areas of the Eastern Highlands (Figs. 2, S2). Moreover, the ^87^Sr/^86^Sr ratios from the volcanic area (Tell Qarassa North) surpass those recorded for the adjacent volcanic regions such as the Golan Heights and the Jebel el Druze^58^, albeit lower than those of the volcanic area of the Black Dessert in North-eastern Jordan^59,60^ (Table S3). Furthermore, most individuals fall within local strontium baseline ratios corresponding to the geological zones of their archaeological sites (Fig. S2). Local ranges of strontium values ratios were obtained for each site from plant and faunal remains collected at archaeological sites and from trimmed datasets of human values (Supplementary Material Text S2).

**Figure 3:**
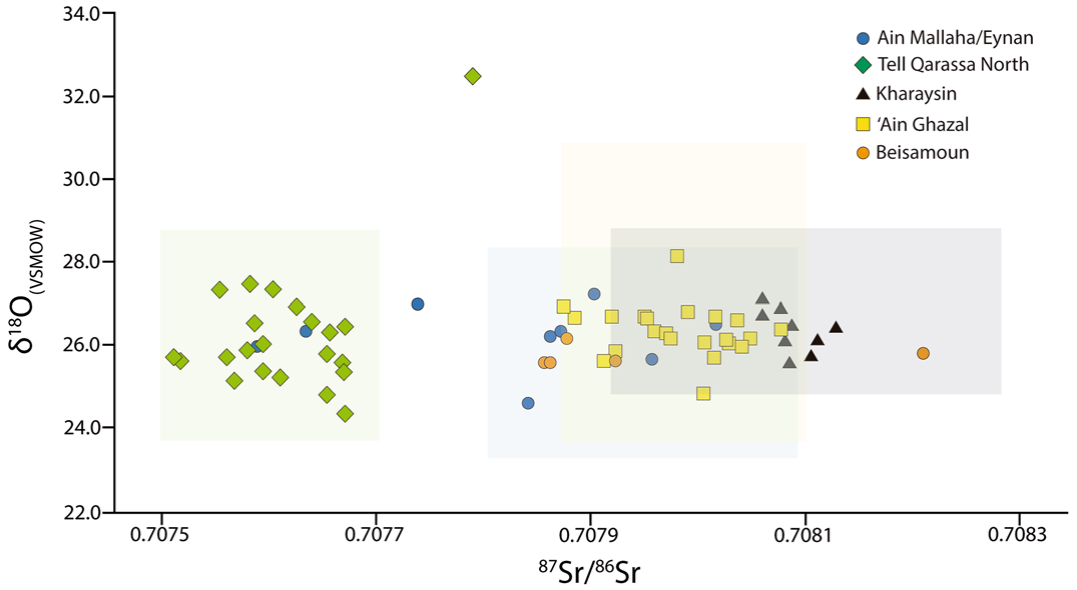
Scatter plot of δ^18^O_carb(VMOW)_ and ^87^Sr/^86^Sr isotope ratios for the 67 individuals from the five archaeological sites of the Southern Levant. The isotope ratios for individual sites are plotted in Fig. S3 (Supplementary Material). The light colour figures mark the Sr local baseline range at each site: blue (‘Ain Mallaha/Eynan and Beisamoun, 0.70782–0.70808); green (Tell Qarassa North, 0.70750–0.70770), yellow (Kharaysin, 0.70792 to 0.70828), and black (‘Ain Ghazal, 0.70787–0.70810).

**Figure 4:**
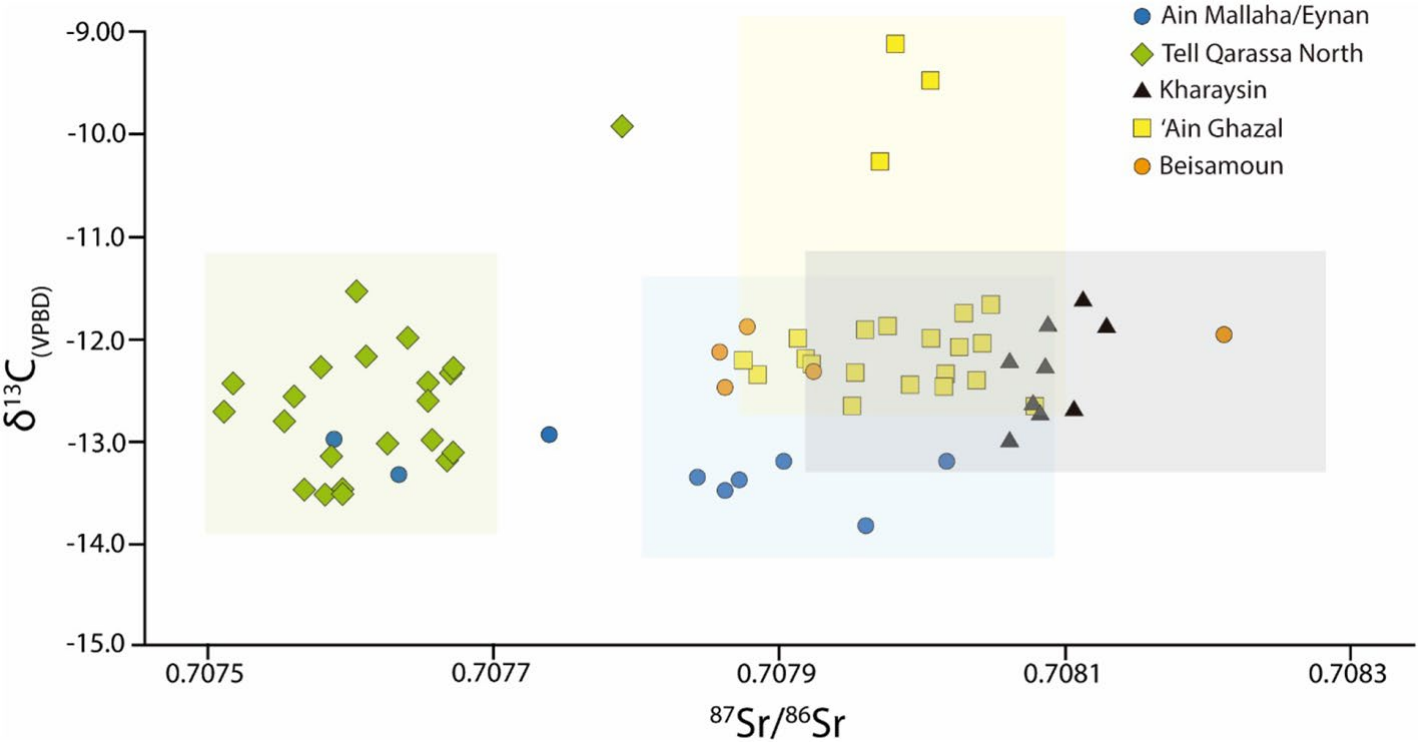
Scatter plot of the δ^13^C and ^87^Sr/^86^Sr isotope ratios of the 67 individuals from the five archaeological sites of the Southern Levant. Isotope ratios for individual sites are plotted in Fig. S4 (Supplementary Information). The light colour figures mark the Sr local baseline range at each site: blue (‘Ain Mallaha/Eynan and Beisamoun, 0.70782–0.70808); green (Tell Qarassa North, 0.70750–0.70770), yellow (Kharaysin, 0.70792 to 0.70828), and black (‘Ain Ghazal, 0.70787–0.70810).

Of the nine individuals from the Natufian site of ‘Ain Mallaha/Eynan that reveal ^87^Sr/^86^Sr ratios (0.70759 – 0.70801; mean = 0.70782 ± 0.0001 2σ), three (33%) fall below the lower end of the local range according to the estimated baseline (0.70782–0.70814; mean = 0.70798 ± 0.00006) and the trimmed dataset of human values (0.70782–0.70808; mean = 0.70794 ± 0.00009 2σ) (Fig. 3; see also Fig. S2, Supplementary Material Text S2). The isotope values of the three outliers from ‘Ain Mallaha/Eynan are in fact consistent with volcanic areas characterised by basalt and pyroclastic outcrops to the north of the Sea of Galilee and the al-Leja region between Southern Syria and Northern Jordan (e.g. Tell Qarassa North)^51,61–64^. Moreover, these outlying strontium isotope ratios are not consistent with data from either the Golan Heights or the highland or coastal areas^29,58^. Interestingly, bone morphometrics among the human remains from ‘Ain Mallaha/Eynan offer no signs of proximity to the coastal Natufian population^38^ implying a connection to the opposite side of the Jordan Valley. The ^87^Sr/^86^Sr values of the bone samples of seven individuals from the site of ‘Ain Mallaha/Eynan published by Shewan^29^ suggest five non-locals among the group of 14 (mean = 0.70788 ± 0.0002 2σ) representing 36% of the overall sample (Fig. S5). The nine individuals examined in this study yielded an average δ^18^O_VSMOW_ value of 26.24 ± 0.25‰ (2σ) of values ranging from 24.67 to 27.25‰, only one offers an outlying signature (24.67‰). This individual also yielded an ^87^Sr/^86^Sr ratio of 0.70784 which is along the lower margin of those considered local at ‘Ain Mallaha/Eynan. Furthermore, carbon isotope analyses from all individuals yielded a mean of − 13.29 ± 0.09‰ (2σ) from a range of − 13.82 to − 12.90‰ (Figs. 4, S3). No outliers were identified among δ^13^C values. These depleted values testify to the dominance of C_3_ foods in the diet of the ‘Ain Mallaha/Eynan residents during the Natufian Period.

The 22 individuals from Tell Qarassa North (EPPNB) reveal ^87^Sr/^86^Sr values between 0.70751 and 0.70779 with a mean of 0.70762 ± 0.00006 2σ (Figs. 3, S2). No local baseline is set for this site as it was impossible to access the area during the study. Human ^87^Sr/^86^Sr trimmed ratios from Tell Qarassa North offer a bioavailable strontium range of 0.70750–0.7077 (mean = 0.70761 ± 0.00005 2σ) (Text S1, Dataset S1). The outlier of this group is in an intermediate position between the volcanic and the limestone areas and it is challenging to pinpoint its origin as similar strontium signatures are recorded in different areas of Southern Levant (Text S2). Oxygen isotope measurements from all individuals yielded an average of 26.29 ± 0.34‰ (2σ) from a range of 24.38 to 32.46‰. δ^13^C values range from − 13.51 to − 9.92 with a mean of − 12.60 ± 0.17‰ (2σ). These values single out only one non-local with an ^87^Sr/^86^Sr signature of 0.70779 (4.5% of individuals) compatible with several locations in the Levant^51,61^. Furthermore, δ^18^O (32.46‰) and δ^13^C (− 9.92‰) values also indicate that this individual is an outlier when compared to the Tell Qarassa North population. Its δ^13^C values suggest a mixed C_3_-C_4_ consumption pattern and may reflect ingesting animals that grazed on C_4_ vegetation (Figs. 4, S3). These data could also be linked to the consumption during childhood of marine resources. Indeed, δ^18^O_carb(VSMOW)_ converted to drinking water yields a δ^18^O_dw(VSMOW)_ value of − 3‰ compatible with that of coastal aquifers^65^. However, the δ^18^O_dw(VSMOW)_ value also lines up with that of the Jordan Rift Valley where bioavailable Sr isotope signatures range from 0.70781 to 0.70798^29,51,58,62^ which, in turn, is close to Sr isotope data of the non-local individual from Tell Qarassa (0.70779).

The ^87^Sr/^86^Sr ratios of the nine individuals from the site of Kharaysin range from 0.70805 to 0.70813 (mean = 0.70809 ± 0.00002 2σ). All fall within local strontium baseline ratios (0.70792–0.70828, mean = 0.70816 ± 0.0001 2σ) and offer no evidence of outliers (Figs. 3, S2, Text S2). These individuals yielded an average δ^18^O_VSMOW_ value of 26.30 ± 0.34‰ (2σ) from a range of 25.57 to 27.11‰. δ^13^C values reveal a mean of − 12.33 ± 0.16‰ (2σ) from a range from − 13.00 to − 11.62‰. The δ^18^O and δ^13^C findings therefore also provide no evidence of outliers at Kharaysin (Figs. 4, S3).

Twenty-two individuals from the site of ‘Ain Ghazal (MPPNB-PPNC) reveal ^87^Sr/^86^Sr average values between 0.70788 and 0.70807 (mean = 0.70798 ± 0.00005 2σ). Local strontium baseline ratios range from 0.7079 to 0.7082 (mean = 0.70815 ± 0.00005 2σ) (Fig. 3; see also Fig. S2, Text S2). The ratios of two cases fall just below the lower end of the local range (0.00003). However, the human trimmed dataset displays a range from 0.70788 to 0.70810 (0.70798 ± 0.00005) meaning they are likely local. Furthermore, one individual overlaps with the dataset of Kharaysin (c. 30 km away) linked to a younger limestone outcrop^66^ suggesting the individual could be non-local (4.5% of all individuals). Otherwise, the δ^18^O values evidence two outliers (mean = 26.36 ± 0.13‰ 2σ from a range of 24.85 to 28.16‰): a non-adult 2 to 4 years of age characterised by the lowest values and an adult with a higher isotopic value, greater than 1.73‰ mean value of all of the individuals from ‘Ain Ghazal. The δ^13^C values from the enamel apatite of this case reveal a mean of − 11.83 ± 0.20‰ (2σ) from a range of − 12.66 to − 9.10‰ (Fig. 4). There are three outliers among the δ^13^C values corresponding to two infants (− 10.25 and − 9.47‰) and an adult (− 9.10‰). The ^87^Sr/^86^Sr ratios of these three falls nonetheless within local strontium baseline ratios (Fig. S3, see also Text S2). Remarkably, the available δ^13^C values of bovid teeth from ‘Ain Ghazal offer evidence that these animals consumed significant amounts of C4 plants^67^. Therefore, the δ^13^C values of these outliers suggest a mixed C_3_–C_4_ consumption pattern and may reflect consumption of animals that grazed on C_4_ vegetation.

Five individuals from Beisamoun reveal strontium isotope ratios ranging from 0.70786 to 0.70821 (mean = 0.70794 ± 0.0001 2σ) (Fig. 3). The strontium local baseline ratio yielded an overall mean of 0.70798 ± 0.00006 (0.70782–0.70814) (Fig. S2) and the human trimmed dataset range from 0.70782 to 0.70808 (0.70794 ± 0.00009 2σ) (Text S2). The group only reveals a single outlier, most likely a non-local, with a different radiogenic signature (20% of all individuals). When regarding the values of Beisamoun and ‘Ain Mallaha/Eynan as a whole, there is still a significant gap between this outlier and the locals from Hula basin where each of the sites are located. They most likely hail from a limestone area, potentially either the eastern or western highlands based on available ^87^Sr/^86^Sr local baseline ratios^29,51,58,60–62,68^. These individuals offer a mean δ^18^O_VSMOW_ value of 25.78 ± 0.22‰ (2σ) from a range of 25.67 to 26.18‰. In addition, δ^13^C values yielded an average of − 12.15 ± 0.9‰ (2σ) from a range of − 12.46 to − 11.89‰ (Fig. 4). No outliers were identified among the δ^18^O and δ^13^C values (Fig. S3).

## Discussion

The current multi-isotope (Sr, O and C) study identified five non-locals (7.5%) among the 67 individuals from the five different sites (Figs. 2, 3, S2, S3). The strontium isotopic ratios serve as evidence of a decrease of non-locals from the Natufian (33%) through the EPPNB (4.5%) and MPPNB (0%) to the Pre-Pottery Neolithic C (6.3%). It is noteworthy that this slight increase in human mobility/migration percentages could be linked to a decline in health and more fragile demographic groups^19^. The non-local number is a minimum estimate due to the fact that the geology of the Southern Levant differs little from north to south, albeit greatly from east to west (Fig. S1), and displacements of individuals with similar ^87^Sr/^86^Sr ratios may go undetected between geologically similar landscapes^61^.

The findings of this study, based on data from ‘Ain Mallaha/Eynan, suggest a significant level of human mobility/migration during the Final Natufian period. Certain exogenous individuals moved to this settlement where they ultimately died and were buried. Noticeably, the archaeological data from the sites of ‘Ain Mallaha/Eynan^69^, Huzuk Musa^70^ and Nahal Ein Gev II^71^ in the Jordan Valley suggest that the Final Natufian Culture (labelled by certain authors as the end of the Late Natufian) featured a sedentary lifestyle. There is indeed straightforward evidence of occupation continuity and growing sedentarization. This is based on the fact that at ‘Ain Mallaha/Eynan^20,69,72^ and other sites^21,70,72,73^ there are features and types of finds that stretch from the later phases of the Late to the Final Natufian, notably constructions with stone foundations and houses that line up in parallel rows, as well as burial grounds and, worth noting, a great proportion of commensal micro-fauna vs wild ones.

These findings differ somewhat from previous strontium isotope analyses of Natufian human populations of the Southern Levant (Table S1)^29^. Strontium isotope data from El Wad B, Kebara, Hayonim Cave, ‘Ain Mallaha/Eynan, Wadi Hammeh 27 and Azraq 18 suggest a pattern consistent with low levels of mobility. This was interpreted as a multi-seasonal activity since mobility is concealed when displacements take place within geological regions marked by similar biological strontium values. This assertion is based on the fact that the isotope signatures of humans and other animal species are identical at both larger and smaller Natufian sites^29^. Diagenesis may have affected both human and faunal samples leading to the narrow range of variation as most results stem from bone remains^43^. However, data between the enamel, bone and plant strontium is consistent. Furthermore, isotopic values are analogous when bone and enamel samples are available for the same individual^29^. Therefore, if the results are within acceptable boundaries, this dataset may serve as evidence of increased territoriality and low human mobility in the Southern Levant during the Natufian Period.

The current results also bolster the idea that Final Natufian settlements in the Jordan Valley are characterised by population aggregates from other areas. Indeed, the epigenetic trait of the palatine torus among the human remains only appears in the Final Natufian layers of ‘Ain Mallaha/Eynan, possibly serving as a marker of newcomer arrival^38^. This being interestingly supported by the appearance of the practice of dental avulsion, absent until now at ‘Ain Mallaha/Eynan but present at other Natufian sites^74^. Human mobility at this site in this timeframe is also attested by short and long-distance exchange networks of goods such as shell beads procured from the Mediterranean and Red Sea coasts^75^, Late Tertiary-Quaternary basalt grinding/pounding tools from outcrops up to 100 km away, and Anatolian obsidian from the northern Levant^76^. It is worth highlighting that the potential origin of the non-locals of the volcanic district of al-Leja (Tell Qarassa North) coincides with the provenance of certain basalts serving to fashion grinding/pounding tools at ‘Ain Mallaha/Eynan^77^. Consequently, human migration and/or mobility may have likewise sustained exchange networks during the Final Natufian, although these materials may be also interpreted as episodic exchanges rather than evidence of prolonged periods of procurement or long distant human mobility^29^.

The current analysis highlights a local origin of most individuals of the Pre-Pottery Neolithic period. Only two, respectively from Tell Qarassa North and Beisamoun, can be placed in the non-local category. It is not possible, on the basis of Sr and O isotope data, to identify the potential area of origin of the Tell Qarassa North case since there is an overlap in the values between the different regions in the Southern Levant. It is plausible nonetheless that the person came either from the Jordanian Rift Valley or from the coastal areas^61,64,68^. Genetic evidence also suggests a limited role of human migration during the PPN period as population structures persisted throughout the emergence of the Neolithic in the Levant, in the Zagros Mountains and in Central Anatolia^24,25,26,29^. It is noteworthy that this pattern is also present at the Early Pre-Pottery Neolithic A site of Körtic Tepe in Anatolia (10 to 9.2 ka cal BC)^78^ where strontium and oxygen isotope analyses imply that most of the population was local^79^. However, it remains clear that human mobility and migration took place at certain moments of this process^37^. Migration is confirmed by the colonisation of Cyprus at the 11th millennium cal BP during the early Pre-Pottery Neolithic^32^. Yet, the lack of genetic evidence hinders understanding whether human migration patterns during the Neolithic transition consisted of short-term massive migration, low-level background gene flow, or both^37^.

The Pre-Pottery Neolithic of the Southern Levant is characterised by the consolidation and expansion of domestication and sedentarism processes. The development of sedentary settlements in this region evolved from small PPNA hamlets with surfaces of about one ha and about 100 inhabitants to extended mega-sites of up to 20 ha in the Middle and Late PPNB (10th to early 9th millennium cal BP). Mega-sites such as ‘Ain Ghazal, Jericho, Beisamoun and Kharaysin had hundreds or even a few thousand residents. This significant development is interpreted as stemming from demographic growth and the aggregation of groups from different areas^14,80^. Population expansion in the Southern Levant must have been linked to a type of agriculture facilitating a fivefold growth rate^17^. The current findings therefore indicate that most of the Pre-Pottery Neolithic individuals were buried around or near their villages. This bolsters the notion that an ongoing population aggregation did not play a significant role in the development of Pre-Pottery Neolithic settlements in the Southern Levant. The findings in fact reinforce the role of demographic growth as the driving force consolidating farming settlements.

This study also specifically stresses the local nature of the 11 individuals represented by the skull cache of Tell Qarassa North (EF-101, Dataset S1)^81^. The crania, broken down into nine adult males, one child and one preadolescent, were arranged into two circular units on the floor of a room. Ten displayed signs of facial mutilation, a practice surely not intended to venerate them. The hypotheses identifying the crania as enemy trophies are unlikely as their isotope data suggest a local origin. Besides, it is noteworthy that the Sr isotope ratio of the non-local individual from Beisamoun reveals a different value than that of the non-locals from the nearby site of ‘Ain Mallaha/Eynan. This suggests differing regions of origin over time from the Natufian to the PPNC. Furthermore, this individual was buried with a wild boar cranium^82^, a rite whose only parallel in the Southern Levant is at ‘Ain Ghazal. The deceased, over 50 years old at the age-of-death, may, due to his non-local origin, have benefitted from a specific social consideration. Furthermore, it is noteworthy that one ‘Ain Mallaha/Eynan non-local dating to the Early Natufian period is a young male laid to rest in a singular pit coated with lime plaster. His burial was later re-opened and the skull displaced slightly in order to accommodate a neonate on his skeletonised neck^83^. It therefore appears to be clear that during the Neolithic transition certain non-locals benefitted from particular burial practices, although perfectly integrated into the common funeral space.

The data gathered by this study also supports the idea of low or local-level human mobility/migration patterns during the Pre-Pottery Neolithic and offers firm, direct evidence of short and long-distance networks (e.g., exchange of Anatolian obsidian)^30,76^ and cultural transmission across the Near East^31,84,85^. Moreover, the exchange networks of the Southern Levant did not require significant migration or long-term migration flows. Ethnohistorical and ethnographic data highlight that low-level migration patterns tend to promote collaborative labour and increase knowledge exchange in communities practicing communal agriculture^86,87^. Therefore, the pattern of low-level mobility/migration observed here could have served to develop sustainable and resilient farming villages during the Neolithic Transition. However, migration and/or mobility of certain individuals must have taken place serving to generate networks integrating the transmission of information, goods and, occasionally, genes. The results therefore suggest that migration or inter-site exchange of individuals was necessary to yield solid, long-term, and resilient interactions during the consolidation of farming settlements in the Southern Levant.

The stable carbon isotope analyses of human dental enamel reveal a prevalence of C_3_ plant and C_3_-plant-consuming animals in the human diet. Moreover, δ^13^C_carb_ values from other periods reinforce the idea of a diet dominated by C_3_-based products^68,88–90^. Enriched δ^13^C_carb_ values among outliers may reflect mixed C_3_–C_4_ consumption patterns or consumption of animals grazing on C_4_ vegetation^56^. The current results therefore underscore a gradual increase of δ^13^C values over time from the Natufian to the MPNNB/PPNC periods (Kruskal Wallis: H = 28.447, df = 4, p < 0.001) (Fig. S4). This cannot be explained by changes in the δ^13^C of atmospheric CO_2_ as this fluctuated less than 0.5‰ over the last 20,000 years^91^. Therefore, this increase must be represented by other environmental or cultural factors. All the individuals exhibited values depleted in ^13^C indicating a reliance on C_3_ foods. However, the δ^13^C values show a greater range during the Early PPNB of Tell Qarassa North which suggests a slightly broader and more variable diet while plant domestication was still in process. δ^13^C values are stable and less variable from the MPPNB to the PPNC after achieving plant domestication. This could indicate that people at Natufian sites and Tell Qarassa North consumed C_3_ resources depleted in ^13^C, in contrast with the C_3_ resources consumed by those from the MPPNB to PPNC. This pattern suggests changes in the diet in the Southern Levant as subsistence practices shifted during the Neolithic transition from a hunter-gathering to agriculture. The archaeobotanical record denotes, for instance, that the first domestic cereals appeared in this region in the EPPNB (11th millennium cal BP) but only became dominant later, from the Middle PPNB at the end of the 10th millennium cal BP^8^. It is noteworthy that δ^13^C values also decreased from the Younger Dryas to the Early Holocene in individuals from the Pre-Pottery Neolithic A site of Körtik Tepe in Anatolia. This variation is interpreted as a consequence of changes in vegetation and the composition and diet of animal species, as well as in a reduction of the spectrum of edible plants^79^. This study’s findings therefore also eventually point to a reduction of plant variability in the human diet during the Neolithic transition.

The time period of this study is marked by a climate shift in the Eastern Mediterranean. Palaeoclimatic data from the period initiated in 15 ka cal BP, based on isotopic analyses of speleothems from the Cave of Soreq^92^, unveil an increase of δ^18^O during the Younger Dryas (13.2 to 11.4 ka) preceded and followed by lower values. This was succeeded by consistent levels of δ^13^C until 11.4 ka, a reduction until 10 ka, and then an increase and fluctuating δ^13^C until the surge linked to the 8.2 ka event. Following the interpretative scheme advanced by Bar-Mathews et al.^92^ for controls on isotope ratios during this speleothem, the periods of lesser δ^18^O were wetter, whereas those of greater δ^13^C are tantamount to temperature increases. It is noteworthy that the current isotopic results appear to reflect this climate shift by an increase in δ^13^C over time from a colder and dryer Younger Dryas to a warmer and wetter early Holocene. Indeed, local individuals from the Final Natufian at ‘Ain Mallaha/Eynan and from the PPNC at Beisamoun, sites in the same area, reveal significant differences. Furthermore, the EPPNB δ^13^C results of this study deriving from the Soreq record also are more variable during the period of warming. Therefore, an alternative explanation of the δ^13^C results is that they reflect a rise of warmer and wetter conditions in the Levant during the Neolithic transition.

## Conclusions

This study offers a glimpse by means of a multi-isotope approach into the role of human mobility and migration during the Neolithic transition in the Southern Levant. It specifically identifies a decrease of the presence of non-local individuals in the period extending from the Final Natufian to Pre-Pottery Neolithic C. The study offers evidence of a significant level of human migration and/or mobility in the Late/Final Natufian period at the site of ‘Ain Mallaha/Eynan that suggests a process of population aggregation in the Jordan Valley. This is consistent with archaeological evidence indicating that Natufian settlements in the Jordan Valley embarked on a process of sedentarization and cultural continuity while simultaneously maintaining short and long-distance networks of interaction and exchange. Furthermore, the data divulge that most individuals from the Pre-Pottery Neolithic Period were local, suggesting that demographic growth was a driving force in the consolidation of farming settlements despite the presence of non-local components. Although the initial stimuli serving to consolidate sedentarism appears to include population aggregation, it was population growth that facilitated the development of farming settlements. The findings therefore bear witness to an amplification of attachment to the land in the Southern Levant as farming became increasingly dominant among the subsistence strategies throughout the Neolithic transition. Finally, the study offers evidence suggesting that complex supra-regional networks of interaction and exchange in the Southern Levant may stem from migration or the movement of a limited number of individuals and not from large-scale mobility.

## Materials and methods

The teeth samples were prepared for analysis at the Isotope Laboratory of the Department of Archaeology of Durham University, United Kingdom. Sr and stable isotope measurements were carried out respectively at the Arthur Holmes Isotope Geology and the Stable Isotope Laboratories of the Department of Earth Sciences of Durham University. Although the choice focused mostly on molars (M2), first molars, premolars and canines were also selected (Table S1). The samples were sectioned with a flexible diamond impregnated cutting disc. The enamel was abraded from the surface with a dental burr discarding the removed material. Any adhering dentine tissue was then removed leaving a 20 mg clean core of enamel for the strontium, oxygen, and carbon isotope analyses. The human enamel samples were prepared for strontium isotope analyses following published procedures^93,94^. Samples were dissolved in 0.5 ml TD 16 M HNO_3_ (where TD refers to Teflon Distilled reagent), then dried and re-dissolved in 0.5 ml TD 3 M HNO_3_. The plant samples were ground in a pestle and mortar and approximately 100 mg was initially dissolved in 3 ml of TD 16 M HNO_3_ on a hotplate at 160 °C. 0.5 ml of 30% H_2_O_2_ was then added to the hot HNO_3_ to bolster the oxidation of organic material before drying. This step was repeated until the solution was clear. Following dissolution, the enamel or plant samples were loaded into columns containing 60 µl of Eichrom Sr-spec resin, the strontium was eluted in 0.4 ml of MQ water and acidified with TD 16 M HNO_3_ to yield a 3% HNO_3_ solution ready for analysis.

Samples for strontium isotope composition analyses were measured by a ThermoFisher Scientific Neptune Multi-Collector ICP Mass Spectrometer (MC-ICP-MS). They were then introduced into a ESI PFA50 nebuliser and a glass expansion cinnabar micro-cyclonic spray chamber, which yields a sensitivity of ~ 60 V ppm^−1^ for Sr at an uptake rate of ~ 90 ml min^−1^. During this study, the average ^88^Sr beam intensity for the enamel samples was set at 23 V, which equates to a Sr concentration of ~ 0.5 ppm, with a minimum and maximum beam size of respectively 10 and 37 V. A single Sr isotope analysis comprises 1 block of 50 cycles with an integration time of 4 s per cycle. The total analysis time was ~ 3.5 min.

Corrections were applied for Kr interferences of ^84^Sr and ^86^Sr deriving from the Ar gas supply and for any Rb interference of ^87^Sr deriving from the sample by respectively monitoring the ^82^Kr, ^83^Kr and ^85^Rb masses. The average ^83^Kr intensity throughout the different analytical sessions was ~ 0.25 mV, which is insignificant considering the Sr beam size (^88^Sr between 10 and 44 V, average of 23 V). The average ^85^Rb intensity was slightly greater and more variable at ~ 0.9 mV (range 0.1–5 mV). Yet like the previous case, given the range in Sr beam size, the typical Rb ^87^Sr/^86^Sr correction was extremely small (< 0.00005) and can be considered accurate at that magnitude.

The enamel samples listed in Table S1 underwent three separate analytical sessions. The average ^87^Sr/^86^Sr value and reproducibility of the isotope reference material NBS987 for each of the sessions is as follows: Session 1: 10–07-18, 0.710267 ± 0.000019 (2σ; n = 10); Session 2: 31–10-18, 0.710254 ± 0.000010 (2σ; n = 10); Session 3: 18–03-19, 0.710235 ± 0.000008 (2σ; n = 10).

The plant samples listed in Table S2 were analyzed in one session during which the average ^87^Sr/^86^Sr value and reproducibility for NBS987 was 0.710244 ± 0.000006 (2σ; n = 6). Data from this study (see Tables S1, S2) were renormalized to an accepted value for NBS 987 of 0.71024.

Carbon (δ^13^C) and oxygen (δ^18^O) isotope ratios were measured from the carbonate (CO_3_) component of tooth enamel following the procedures published by Bentley et al.^95^. Approximately 2 mg of powdered sample was placed in 99% ortho-phosphoric acid for 2 h at 70 °C. The resultant helium and CO_2_ gas mixture was then separated and analyzed for isotopic data via a Thermo Scientific Gasbench II interfaced with a Thermo Scientific MAT 253 gas source mass. Duplicate analysis of 11 samples yielded a precision with a mean difference of 0.17‰ for δ^13^C and 0.18‰ for δ^18^O. Repeated analysis of both international reference materials (NBS 18, n = 14, IAEA-CO-1, n = 14, LSVEC, n = 14) and internal laboratory standards (DCS01, n = 30 and DOBINS, n = 9) yielded analytical reproducibility greater than 0.10‰ (s.d) for δ^13^C and 0.15‰ (s.d) for δ^18^O. All values were normalized to the accepted values of + 2.49‰ and − 46.60‰ for δ^13^C, and − 2.40‰ and − 26.70‰ for δ^18^O, for IAEA-CO-1 and LSVEC, respectively. Furthermore, the oxygen isotopic composition of ancient drinking water was estimated from enamel carbonate data by the equation of Chenery et al.^55^ (δ^18^O_Drinking water_ = 1.590 × δ^18^O_VSMOW (carbonate)_ − 48.63).

## Supplementary Information

Supplementary Information 1.

Supplementary Information 2.

## Data Availability

All data needed to evaluate the conclusions in the paper are present in the paper and/or the Supplementary Materials. Correspondence and material related to this paper may be requested from Jonathan Santana (jonathan.santana@ulpgc.es).
