# Supplementary material for: Multi-isotope evidence of population aggregation in the Natufian and scant migration during the early Neolithic of the Southern Levant
Source: Sci Rep. 2021 Jun 4;11:11857. doi: 10.1038/s41598-021-90795-2 (PMC8178372; doi:10.1038/s41598-021-90795-2)
Supplement: Supplementary file 1 — Supplementary Information 1. [file 41598_2021_90795_MOESM1_ESM.docx]

**Supplementary Material**

**Multi-isotope evidence of population aggregation in the Natufian and scant migration during the early Neolithic of the Southern Levant**

Jonathan Santana^*^, Andrew Millard, Juan J. Ibáñez-Estevez, Fanny Bocquentin, Geoffrey Nowell, Joanne Peterkin, Colin Macpherson, Juan Muñiz, Marie Anton, Mohammad Alrousan, Zeidan Kafafi

^*^Jonathan Santana

Email: [jonathan.santana@ulpgc.es](mailto:jonathan.santana@ulpgc.es)

**Summary**

**Text**

S1 Archaeological sites 2

S2 Geology of the Southern Levant and multi-isotope variation 5

**Figures**

S1 Simplified geology of the Southern Levant and location of sampled sites 7

S2 Scatter plots of the δ18O and 87Sr/86Sr ratios by site 8

S3 Scatter plots of the δ13C and 87Sr/86Sr ratios by site 9

S4 Box plot of the δ13C values ratios according to chronological periods 10

S5 Scatter plot of the 87Sr/86Sr ratios for the ‘Ain Mallaha/Eynan sample 11

**References** 12

Dataset S1 (separate file).

Table S1 List of sampled human teeth and results of isotope analysis

Table S2 Local Bioavailable ^87^Sr/^86^Sr ratios for each archaeological site

Table S3 Local Bioavailable ^87^Sr/^86^Sr ratios for other locations in Southern Levant

Text

S1 Archaeological sites

The dental material sampled for the current study was collected at five different sites of the Southern Levant. It consists of enamel samples from 67 humans from primary and secondary burials from the Natufian, Early Pre-Pottery Neolithic B, Middle Pre-Pottery Neolithic B and Pre-Pottery Neolithic C periods, a timespan that in the Near East englobes the Neolithic transition. The corpus can be broken down into 9 from ‘Ain Mallaha/Eynan, 22 from Tell Qarassa North, 9 from Kharaysin, 22 from ‘Ain Ghazal, and 5 from Beisamoun (Fig. 1, Fig. S1). Further information as to the samples is detailed in Dataset 1 (Table S1). Sex and age-of-death are times not reported systematically due to the lack of diagnostic skeletal regions of human remains, impeding isotope analyses based on sex or age groups. The main goal of this study is to identify allochthonous individuals so as to delve into the question of human mobility and residential behavior. Direct radiocarbon dating of the samples was not attempted because bone collagen is poorly preserved at Near Eastern prehistoric sites^1^ and our attempts to extract it were unsuccessful. The chronological framework was assigned according to archaeological contextual information from the five sites that along with their environmental contexts are briefly described below.

*‘Ain Mallaha/Eynan /Eynan*

‘Ain Mallaha (Arabic) or Eynan (Hebrew) is an open-air settlement dated to the Natufian period. It is perched on an elevation (+72 m a.s.l) near the northern limit of the Jordan Rift Valley. It is in the Hula Basin along the eastern slopes of the Upper Galilee adjacent to the Naftali Mountains. The basin is a corridor 25 km long and 8 km wide between the Galilee and the Golan Heights formed during the Plio-Pleistocene and collects sedimentary materials from the Jordan River that runs through it^2,3^. The river flows through the calcareous Naphtali Mountains to the west and the basaltic Golan Heights to the east^4,5^. The western mountains are formations from the Cenomanian-Early Cretaceous period while the Golan Heights is Quaternary volcanic. The Hula Basin is delimited by a gradual transition toward the elevated Beqa‘a Valley to the north. Lacustrine sediments in this area indicate the existence at one time of a freshwater lake. There are also both Cretaceous and Jurassic, and Eocene and Pliocene strata^6^. The southern border is marked by basaltic hills referred to as the basalt ‘plug’ from Upper Pliocene and Pleistocene^7^. The sedimentary sequence of the Hula Basin comprises conglomerates, chalk, clay, peat and basalt originating from several sources. The basalt soil is from the eastern mountains, *terra rossa* and grey-brown soil is from the eastern mountains, and greyish-white soils are limestone deposits within the plain^8^. ‘Ain Mallaha/Eynan's geological features contain calcareous marl, a mixture of calcareous materials and *terra rossa* from the Naphtali slopes. The settlement is near two major freshwater springs and the north-western shore of the Hula Lake, a freshwater lake artificially drained in the 1950s to be used for agriculture. It is a shallow basin (5.3 km long, 4.4 km wide, 12–14 km^2^) with an average depth of 2 m.

‘Ain Mallaha/Eynan falls within the Mediterranean woodland phytogeography zone characterised by a naturally high biodiversity^9,10^. The area is dominated by nut and fruit bearing trees such as evergreen oak (Quercus calliprinos), deciduous pistachio (*Pistacia palaestina*), deciduous oak (*Q. ithaburensis*), olive (*Olea europaea*) and almond (*Amygdalus communis*). ‘Ain Mallaha/Eynan also benefited from a rich ecosystem due to the springs, streams and the perennial wetlands of the Hula Lake^4^. Early and Late Natufian phytoliths suggests a dominance of woody plants in the Early Natufian to non-cereal grasses in the Late Natufian^11^. Present-day climate conditions are semiarid with an average annual rainfall of 400-500 mm. The mountainous zones retain higher annual rainfall with winter rains, depending on the elevation, between 600 and 1300 mm^9^.

‘Ain Mallaha/Eynan was explored during archaeological missions between 1955 to 2005 under different directors and teams (1955–1961, J. Perrot; 1972–1976, J. Perrot, M. Lechevallier, and F. Valla; and 1996–2005, F. Valla and H. Khalaily)^12^. The archaeological record places the occupation of ‘Ain Mallaha/Eynan during the three Natufian cultural phases (Early, Late and Final Natufian) spanning c. 14,300 cal BP (Early Natufian) to c. 11,500 cal BP (Final Natufian)^13,14^. The presence of dwellings, burials, heavy basalt pounding tools and commensal faunal links to the site from an early phase of the transition to a sedentary way of life among hunter-gatherers prior to the introduction of real farming^15,16,17,18,19^. The Early Natufian phase (c. 15,000-13,000 cal BP) is marked by a cluster of larger dwellings and with burials under their floors^20^. The subsequent Late Natufian (c. 13,000-12,000 cal BP) sees a reduction in the size of dwellings and successive collective burials. The Final Natufian phase (c. 12,000-11,500 cal BP) features small houses^21^. The archaeological evidence places this final phase as lasting until the end of the Younger Dryas^14,22^.

*Tell Qarassa North*

Tell Qarassa North is a settlement along the shore of a paleolake in the volcanic district of al-Lejá. Fieldwork here was conducted in 2009 and 2010 by a Spanish team^23^ as part of the Syrian French-Spanish archaeological research project exploring the desiccated lake of Qarassa^24,25^. The tell is at 750 m ASL and 25 km to the west of the Jabal al-Arab Mountains. The site is along the southern edge of a Pleistocene lava field near Pliocene basaltic materials. It is to the east of the ancient lake from the Late Pleistocene to the Mid-Holocene^24,26,27^. Archaeobotanical finds places it within the Irano-Turanian and Mediterranean woodland phytogeographical regions. The local vegetation included woodland-steppe components and riparian taxa while Mediterranean Quercus woodlands and coniferous forests dominated the mountain area of the Jabal al-Arab to the east^28^. Present-day climate conditions are cold winters and hot summers, and a mean annual precipitation of 350-400 mm^29,30^.

The archaeological evidence indicates occupations from the Early PPNB, Pottery Neolithic and the Chalcolithic. The two areas (Zone 1 and Zone 2) with the most evidence of Early PPNB levels were excavated for the most part between 2009 to 2010^23,27^. The archaeobotanical finds of the two zones provided direct morphological evidence of domesticated cereals in the Early PPNB^31,32^. Among the finds are ground stone tools such as saddle querns and mortars, imported materials such as obsidian^26^, anthropogenic figurines^33^, and faunal remains^27^. Several structures from Zone 1 date to the mid-11^th^ millennium cal BP. The two phases of occupation are separated by a level of fire and destruction^34^. In the second half of the 11^th^ millennium (Beta-272103: 9320±50 BP, 10683 to 10303 cal BP 2σ; Beta-274098: 9300±50 BP, 10623 to 10292 cal BP 2σ) the site saw new buildings and a burial ground. The funerary features comprise five primary tombs enclosing five individuals and five secondary deposits of eight individuals. Most of the primary burials were opened long ago after the time of body decay to remove the skull and certain limb bones. These remains, probably serving for rituals, were then deposited as secondary offerings^35^.

A trench was sunk into the slope of Zone 2 revealing Chalcolithic, Pottery Neolithic and Early PPNB phases. The Early PPNB level is marked by curved stone wall dwellings dated to the second half of the 11^th^ millennium cal BP (CNA1074: 8960±45 BP, 10231 to 9909 cal BP 2σ; CNA1059: 9110±35 BP, 10375 to 10119 cal BP 2σ). The dating of an earlier construction phase is similar (CNA1058: 9010±45 BP, 10248 to 9925 cal BP 2σ; CNA1060: 9145±40 BP, 10485 to 10228 cal BP 2σ). Two rooms of this level were unearthed. One with an oval shape and a door at the northern end of its longer axis contained a skull cache. Two of the three flagstones laid on the floor held the crania. The assemblage consisted of two groups arranged in two circles. They correspond to 12 young males, one infant and one adolescent. The postcranial remains of another adult were placed near to the door at the room's north end. The facial skeletons of the crania were intentionally fractured and discarded before placing them in the room. The cache is thought to represent disaffected individuals and not a rite of ancestor veneration^36^. Those retained for the current study are from the burials of Zone 1 and the skull cache of Zone 2.

*Kharaysin*

Kharaysin (Quneya, Zarqa) is a vast settlement (c. 25 ha) in the Jordanian Highlands discovered in 1984 by Hanbury-Tenison and colleagues during a survey of the Jerash Region^37^. Systematic fieldwork from 2015 to 2019 identified four levels of occupation in four zones: 1) Zones A and B: a Late PPNA phase from the outset of the 11^h^ millennium cal BP; 2) Zone B: an Early PPNB phase from the second half of the 11^th^ millennium cal BP; 3) Zones A and C: a Middle PPNB phase from the outset of the 10^th^ millennium cal BP; and 4) Zone D: a Late PPNB phase from the end of the 10^th^ to the beginning of the 9^th^ millennium cal BP^38,39,40^.

The human samples selected for this study (Dataset 1, Table S1) are from the MPPNB phase of Zone A (CNA4068: 8756±50 BP, 10111 to 9549 cal BP 2σ), an area is characterized by rows of parallel rectangular houses with lime plastered floors that form east-west terraces perpendicular to the slope^39^. The burial grounds of this zone, made up of 10 depositions, cut through the backfill of an abandoned house. The features consist of seven primary burials (six single and one double), one secondary burial, one skull cache, and a single disturbed burial. The stratigraphic sequence indicates that the cemetery served for several years during the MPPNB. Its more notable finds include a stone vessel and a cache of Nahal Hemar knives. It is noteworthy that two clusters of flint anthropomorphic figurines were unearthed in the surroundings suggesting a link to the funerary rituals. The multi-stage burials and the ritual paraphernalia suggest recurrent funerary rituals^41^.

*‘Ain Ghazal*

‘Ain Ghazal is a major Neolithic settlement situated in the surroundings of Amman (Central Jordan). The site is within the foot and toe slopes in the Wadi Zarqa River Valley which during the early Holocene flowed permanently as a stream^42^. It was discovered in the 1970s during construction of the Amman-Zarqa motorway and excavated between 1982 and 1998^42,43^. ‘It is at 725 m ASL on the edge of the Mediterranean woodland phytogeography zone near to the steppe boundary dominated by the Irano-Turanian ecotone^44,45^. This is a semi-arid location with a mean annual precipitation of about 270 mm^46^ supporting a thermophilous steppe forest^44,45^.

The settlement is one of the Southern Levant's mega-sites^42,47^ characterized by four major phases: a) Middle PPNB (MPPNB, 10.1–9. ka cal BP), Late PPNB (LPPNB, 9.5–8.9 ka cal BP), PPNC (8.9–8.4 ka cal BP), and Yarmukian Pottery Neolithic (8.4–7.5 ka cal BP). Its initial small village in the MPPNB period covering a surface of 4 to 5 ha grew to 12-13 ha by the end of the LPPNB period^42^. It is marked by elaborate architecture, public buildings, burials, and symbolic finds. Its residents relied on a wide set of domestic and wild plants such as wheat, barley, peas, lentils, chickpeas, figs, almonds and pistachios^42,48,49^. Animal exploitation strategies included intensive hunting, experimentation with animal husbandry, and caprine harvesting^50,51,52,53^.

*Beisamoun*

The Pre-Pottery Neolithic site of Beisamoun is along the western edge of the Hula Basin in the upper Jordan Rift Valley (see Hula Basin description above). It is a privileged setting between two freshwater springs in proximity of the north-western shore of the Hula Lake. It is near the Natufian site of ‘Ain Mallaha/Eynan (see above) and dated to the Pre-Pottery Neolithic B and C^54,55,56^. The site was discovered in 1955 during the construction of pisciculture features. Surface surveys and mapping of its walls was conducted during the 1960’s and 1970’s by A. Assaf and a team directed by M. Lechevallier^54^. House 150 brought to light in 1972 yielded a very rich assemblage including two plastered skulls^54^.

Further excavations were carried out by F. Bocquentin and H. Khalaily from 2007 to 2016^55,56^ confirming that Beisamoun was occupied during the MPPNB (first half of the 10^th^ millennium cal BP), the LPPNB (second half of the 10^th^ millennium cal BP) and the PPNC (first half of the 9^th^ millennium cal BP)^57^. During the Late PPNB, it was one of the largest sites in the Southern Levant, extending over an estimated area of 10 ha^54^ and considered as part of the mega-site phenomenon characterized by sites such as ‘Ain Ghazal, Jericho and Kharaysin^55,57^. Its architectural features and burials coupled with finds of lithic artefacts and fauna indicate a dense occupation. The superposition of numerous phases of construction devoid of hiatus throughout the Late PPNB to PPNC transition of c. 9200-9000 cal BP^56,58^. Ultimately, the site was abandoned at the end of the Pre-Pottery Neolithic period at c. 8400-8200 cal BP. An Early Pottery Neolithic occupation, with no trace of superposition between the Late PPNB to PPNC phases, is also identified further west at Beisamoun West^55,59^.

S2 The geology of the Southern Levant and multi-isotope signatures

*Strontium isotopes*

The geology of the study area is divided in five main zones: Jordan Rift Valley, Eastern and Western Highlands, Azraq Basin, Golan Heights and the basalt outcrops from southern Syria (Fig. S1). The sites of ‘Ain Mallaha/Eynan and Beisamoun are in the Hula Basin in the upper area of the Jordan Rift Valley, ‘Ain Ghazal and Kharaysin in the Eastern Highlands (Jordanian Highlands) and Tell Qarassa North in the volcanic district of the al-Lèja in southern Syria. Bioavailable ^87^Sr/^86^Sr ratios from modern and archaeological samples served to establish the local range of each site. Furthermore, alternative estimates of local ranges were carried out using intra-site human ratios by means of trimmed datasets^60,61^. Outlier values beyond the major clusters were removed to offer a more normal distribution and improve the extent of local ratios^61^.

The Jordan Rift Valley is a geological depression from the Miocene encompassing the Jordan River, the freshwater Sea of Galilee in the north and the Dead Sea in the south. This area is mainly characterized by Quaternary gravels, mudstone, and sandstone^62,63^. Modern and archaeological fauna from this zone reflect ^87^Sr/^86^Sr ratios between 0.70782 and 0.70803^62,64,65,76^. Water samples from the Dead Sea yield ^87^Sr/^86^Sr ratios between 0.70802 and 0.70803^67,68^. In addition, bioavailable ^87^Sr/^86^Sr ratios of archaeological samples from the Dead Sea Plain range from 0.70790–0.70840^69^. ‘Ain Mallaha/Eynan and Beisamoun are in the Hula Basin in the upper area of the Jordan Rift Valley in Upper Galilee, an area of basalt, *terra rossa* and cretaceous limestone soils. Bioavailable ^87^Sr/^86^Sr ratios range from 0.70760 to 0.70820 based on modern plants and archaeological faunal dental enamel (Table S2). The dataset includes data published by Shewan^66^. However, this study reevaluated the local ^87^Sr/^86^Sr range removing the outliers: 0.70782 - 0.70814; mean=0.70798 ± 0.00006. Furthermore, a local range of 0.70782 - 0.70808 (0.70794 ± 0.00009 2σ) was estimated using the trimmed dataset (Fig. S2).

Tell Qarassa North is located at the al-Lèja in the northern area of Harrat Ash Shamah, the largest volcanic plateau of Arabia. It covers large parts of southern Syria extending about 500 km from the Golan Heights into Jordan and Saudi Arabia^70,71^. The Golan Heights and the surrounding Sea of Galilee are characterized by volcanic sediments from Pliocene and Pleistocene basalts. This area yielded ^87^Sr/^86^Sr ratios ranging from 0.70470 to 0.70690 due to the presence of young basalts^64^. The area of Tell Qarassa North reflects of three episodes of volcanic activity: Miocene, Pliocene and Quaternary^70,71,72^. No bioavailable ^87^Sr/^86^Sr ratios are available. Geological ^87^Sr/^86^Sr ratios nonetheless display greater radiogenic values than those of the Golan Heights^73^. The local baseline was estimated using the trimmed dataset of human values yielding an ^87^Sr/^86^Sr range of 0.70750 – 0.70770 (mean= 0.70761 ± 0.00005 2σ) (Fig. S2).

‘Ain Ghazal and Kharaysin are located on the limestone units of the Jordanian Highlands (Eastern Highlands). This area is an average of 1100 m above the Jordan Rift Valley and primarily comprises Cretaceous limestone bedrock with Quaternary sediments and undifferentiated basalts^74,75^. There is a thin corridor of mountains just east of the Jordan Rift Valley marked by Paleozoic, Jurassic limestone marls and dolostones to the north and sandstones from the Paleozoic and Lower Triassic to the south^63,74^. Paleozoic and Mesozoic outcrops also appear in the Rift Valley drainage system^63^. ^87^Sr/^86^Sr ratios of rodent enamel in this sector range from 0.70792 to 0.70810^62^. Bioavailable ^87^Sr/^86^Sr ratios reflect a range from 0.70801 to 0.70854 based on modern plants and archaeological rodent enamel^62,76^ (Table S3). Modern plants ^87^Sr/^86^Sr ratios at Kharaysin reflect a local baseline from 0.70792 to 0.70828 (0.70816 ± 0.00012) (Table S2). Bioavailable ^87^Sr/^86^Sr ratios of ‘Ain Ghazal reflect a range of 0.70801 to 0.70854 based on modern plants and the dental enamel of archaeological fauna (Table S3). The revision by this study of the local range (0.70787-0.70810) yielded a mean of 0.70798 ± 0.00005 (Fig. S2).

The highland system west of the Jordan Rift Valley (Western Highlands) consists of limestones from the Upper and Lower Cretaceous combined with dolostones and marls^67^. Modern samples (plant and animal) and archaeological fauna offer ^87^Sr/^86^Sr ratios ranging from 0.70801 to 0.70836^64,66,69,78^ (Table S3). Modern bioavailable ^87^Sr/^86^Sr ratios of the more northern Western Highlands yielded ^87^Sr/^86^Sr ratios between 0.70790 and 0.70840^64^. The Golan Heights and the surrounding Sea of Galilee, to the north of the Jordan Rift Valley, offer lower ^87^Sr/^86^Sr ratios ranging from 0.7047 to 0.7069 due to the presence of young basalts^64^.

The Azraq Basin, farther east of the Eastern Highlands, includes Quaternary gravel plains that marked by several bedrocks: Early Tertiary limestone plains to the west/south-west, Pliocene Basalt boulder fields to the north/north-eastern sector, and the Cretaceous limestone Jordanian Highlands to the west^74,75^. Bioavailable ^87^Sr/^86^Sr ratios yield a range of 0.70807 to 0.70828 in the Tertiary limestone, 0.70803 to 0.70816 in the Quaternary gravels and 0.70764 to 0.70787 in the basalts^62,77^ (Table S3).

*Oxygen isotopes*

The meteoric water oxygen isotope (δ^18^O_w(VSMOW)_) ratios of the Southern Levant vary from north to south and east to west due to the interplay of temperature, precipitation, elevation and distance from the Mediterranean coastline^78,79^. Meteoric water enriched in ^18^O is also observed in areas of low elevation such as the Mediterranean coastal plain and the Jordan Rift Valley^80^. There is a gradual depletion of ^18^O in meteoric water from the coastline eastwards and from the Jordan Rift Valley to the Highlands^81^. Indeed, this composition, according to the δ^18^O values collected from modern rainfall stations^82,84^, fluctuates approximately between -0.21 to −0.15‰ according to the to the cooler temperature of every 100 m increment of elevation. The Azraq Basin also reflects meteoric water enriched in ^18^O due to brief periods of rainfall coupled with events of rapid evaporation^62,84^.

*Carbon isotopes*

Plants absorb atmospheric carbon in one of two ways and are categorized as relying on either C_3_ or C_4_ photosynthetic pathways^85^. C_3_-plants dominate the landscape in the Southern Levant while the distribution of C_4_-plants increases in the more arid regions^86^. The average δ^13^C values for modern plants range from -30.8‰ to -22.5‰ for C_3_ plants and -11.9 to -14.5‰ for those of C_4_^76,77,79,87^. This area includes the Mediterranean and Irano-Turanian biomes, although the first is that which predominates the sites of this study. Each of the biomes are depleted of ^13^C during the late winter, spring, and early summer due to reduced C_4_ biomass and high-water availability promoting the growth of these plants. Dry seasons also produced an increase in the δ^13^C of C_3_ taxa by as much as 7.7‰ due to wet season growth^87,88^. Fractionation between diet and human enamel carbonate yields values around +11-12‰, so that human δ^13^C_ap(VPDB)_ values of -12‰ reflect a diet dominated by plant-consumption of C_3_ plants, whereas the value of -1‰ suggests C_4_ resources^89^. δ^13^C_(VPDB)_ values of enamel carbonate, in turn, reflect a total diet reflecting an intake of carbohydrates, lipids, and proteins^90,91^. Therefore, significant shifts in δ^13^C values from human enamel apatite can also serve to identify different geographical areas or shifts in consumption patterns during childhood resulting from practices of subsistence.

Fig. S1. Simplified geology of the Southern Levant and the location of the sites under study (adapted after Sneh et al.^63^, Ponicarov et al.^72^, and Bender^74,75^). Bottom right corner: simplified map of the main geological zones of Southern Levant; 1: Coastal areas; 2: Western Highlands; 3: Jordan Rift Valley; 4: Eastern Highlands; 5: Golan Heights; 6: Hula Basin; 7: Basalt area; 8: Azraq Basin. The figure was generated using Adobe Illustrate CC 2019 (<https://www.adobe.com/cn/products/illustrator.html>).

­­­
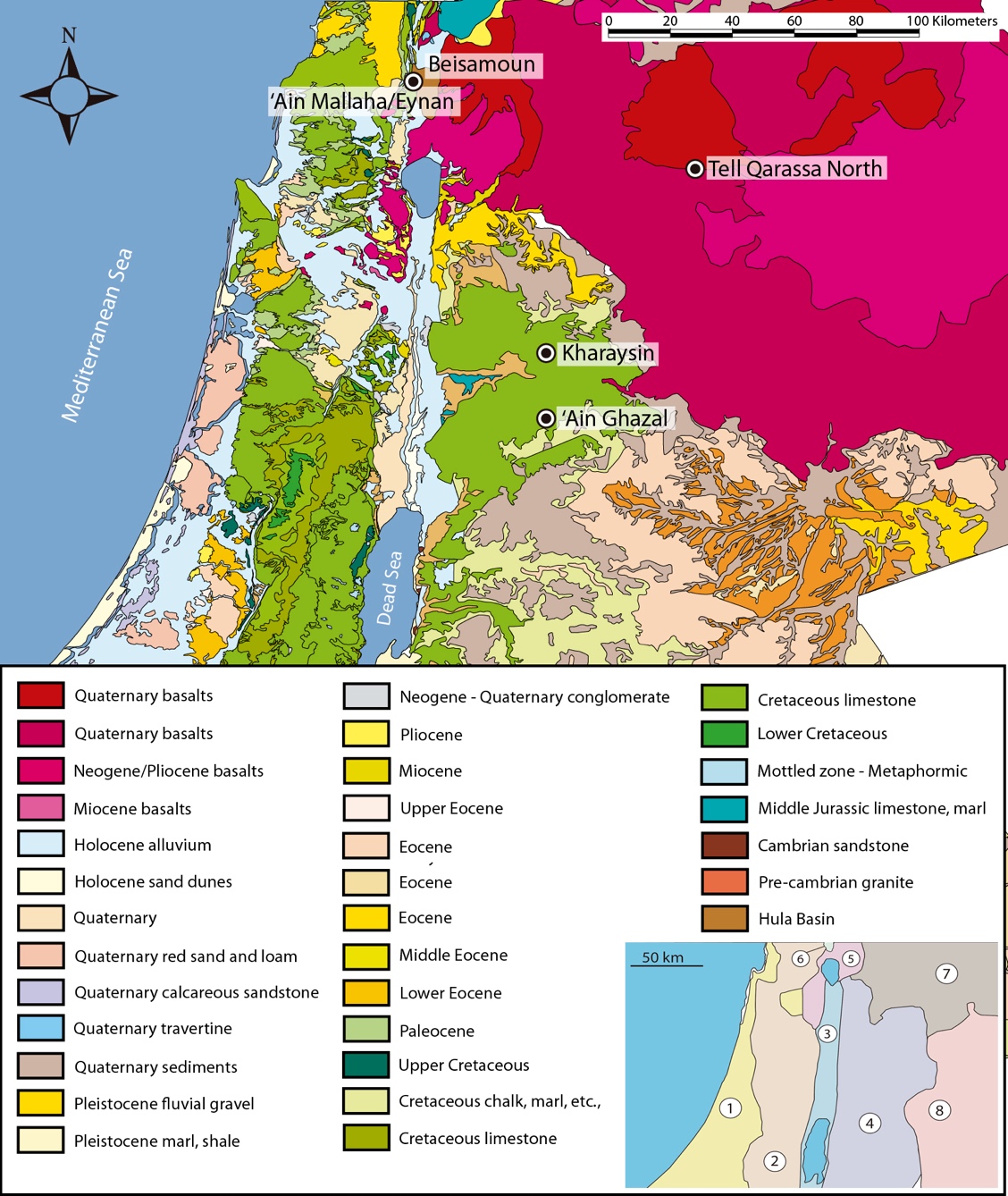


**Fig. S2.** Scatter plots of the δ^18^O and ^87^Sr/^86^Sr ratios by site. The light blue bands mark the Sr local baseline range at each site: ‘Ain Mallaha/Eynan and Beisamoun, 0.70782 - 0.70808; Tell Qarassa North, 0.70750 – 0.70770; Kharaysin, 0.70792 to 0.70828, and ‘Ain Ghazal, 0.70787-0.70810.


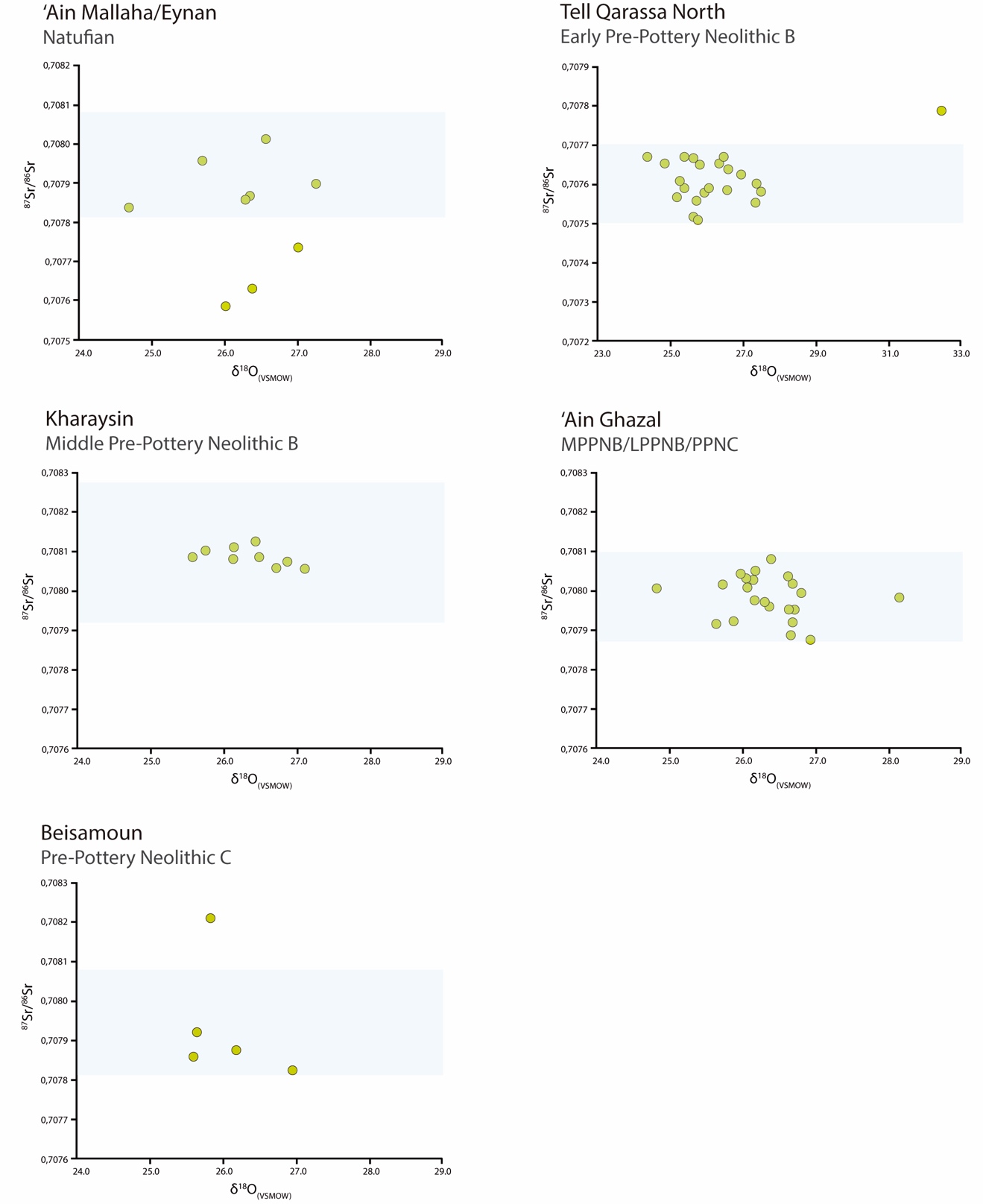


**Fig. S3.** Scatter plots of the δ^13^C and ^87^Sr/^86^Sr ratios by site. The light blue bands mark the Sr local baseline range at each site: ‘Ain Mallaha/Eynan and Beisamoun, 0.70782 - 0.70808; Tell Qarassa North, 0.70750 – 0.70770; Kharaysin, 0.70792 to 0.70828, and ‘Ain Ghazal, 0.70787-0.70810.


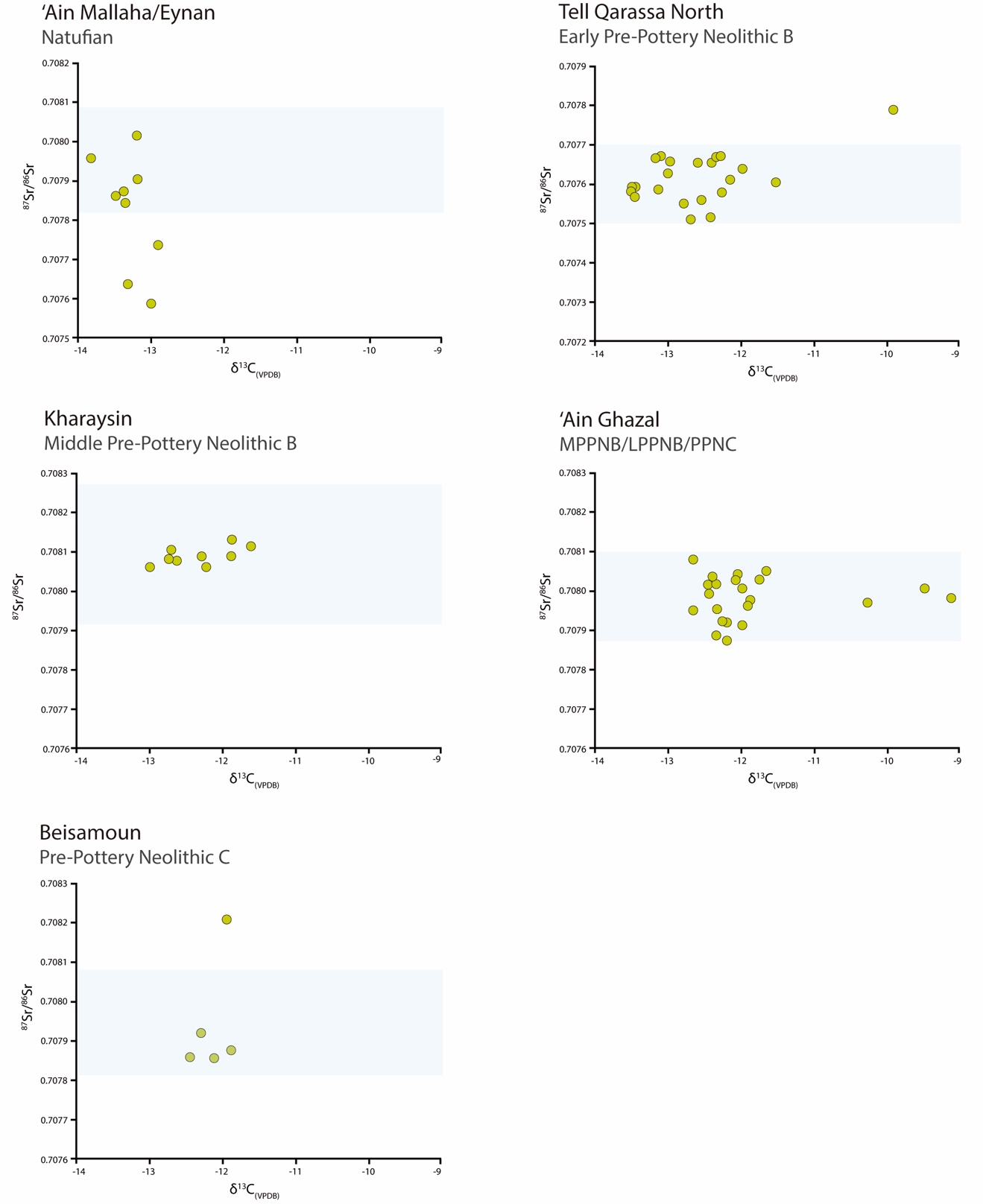


Fig. S4. Boxplots of the δ^13^C values ratios according to chronological periods.


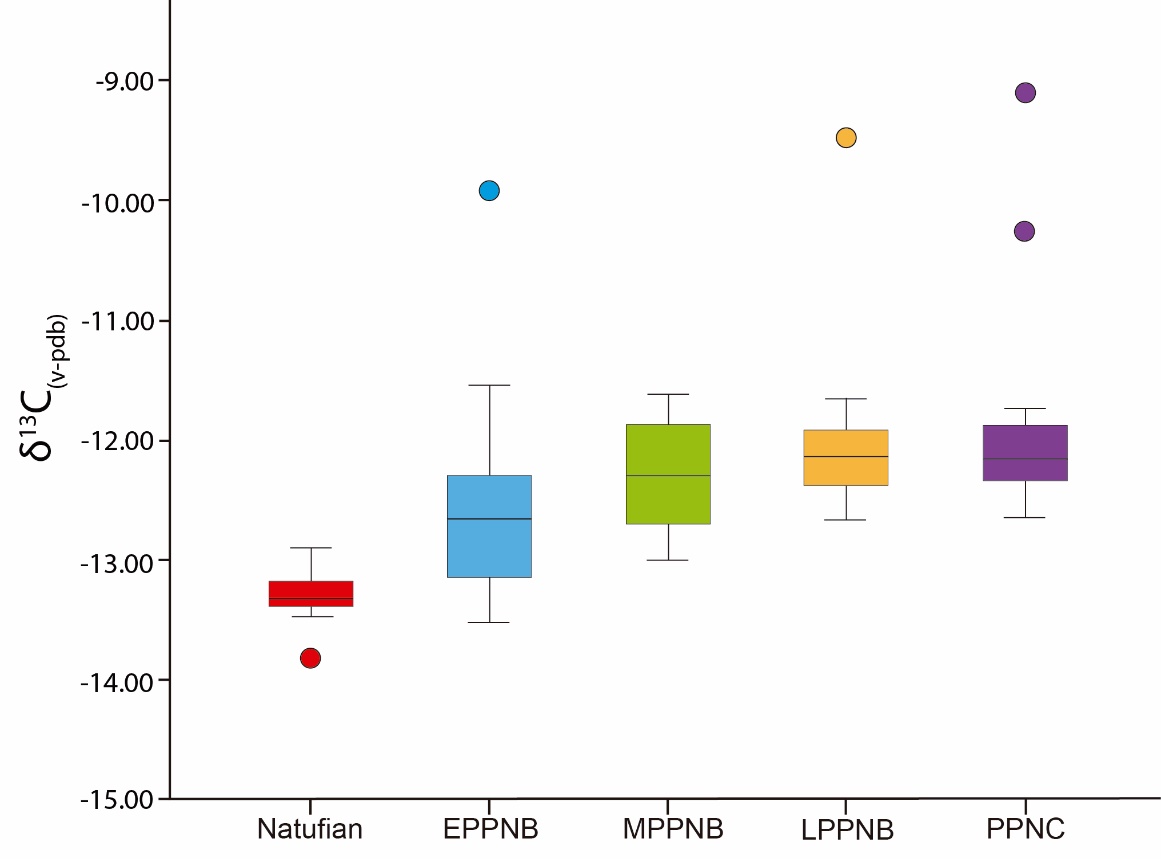


**Fig. S5.** Scatter plot of the ^87^Sr/^86^Sr ratios for the ‘Ain Mallaha/Eynan samples (Sr local baseline range: 0.70782 - 0.70808).


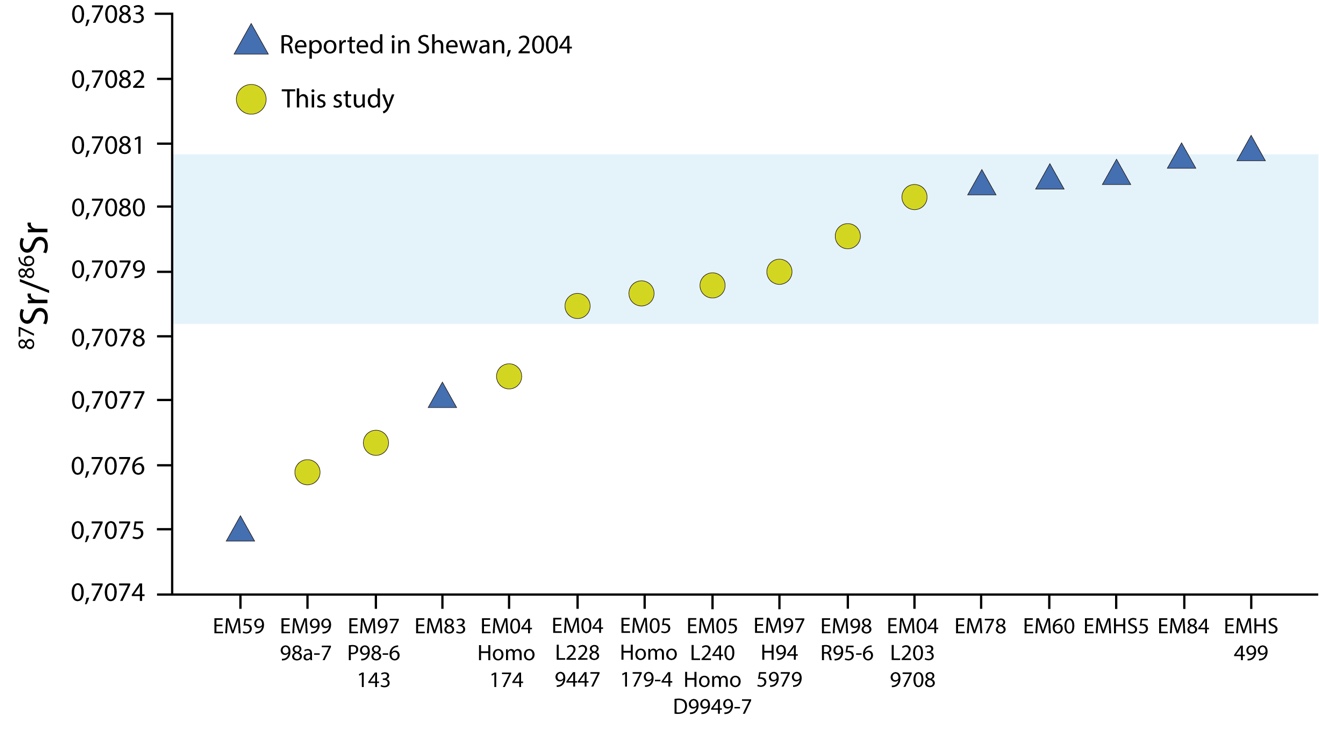


­­­

**SI References**

1. Weiner, S. & Bar-Yosef, O. States of preservation of bones from prehistoric sites in the Near East: a survey. *J. Arch. Sci.* **17**(2), 187-196 (1990).
2. Garfunkel, Z. Internal structure of the Dead Sea leaky transform (rift) in relation to plate kinematics. *Tectonophysics* **80**, 81–108 (1981).
3. Sneh, A. & Weinberger, R. Geology of the Metulla quadrangle, northern Israel: implications for the offset along the Dead Sea Rift. *Isr. J. Earth Sci.* **52**, 123–138 (2003).
4. Dimentman, C., Bromley, H.J. & Por, F.D. *Lake Hula: reconstruction of the fauna and hydrobiology of a lost lake* (The Israel Academy of Sciences and Humanities, Jerusalem, 1992).
5. Shtober-Zisu, N. Geological and geomorphological settings*.* In *An early Pottery Neolithic occurrence at Beisamoun, the Hula Valley, Northern Israel: the results of the 2007 salvage excavation* (Ed. Rosenberg, D.) 15–18. (Archaeopress, Oxford, 2010).
6. Horowitz, A. The Jordan Rift Valley (CRC press, Boca Ratón, 2001).
7. Goren-Inbar, N. et al. Evidence of hominin control of fire at Gesher Benot Yaaqov, Israel. *Science* **304**(5671), 725-727 (2004).
8. Zohary, M. & Orshanskyand, G. The vegetation of the Huleh Plain. *Palestine J. Bot.* **4**, 90-105 (1947).
9. Weinstein-Evron, M. The paleoecology of the early Wurm in the Hula basin, Israel. *Paléorient* **9**(1), 5-19 (1983).
10. Ashkenazi, S. Reconstruction of the habitats in the ecosystem of the final Natufian site of ‘Ain Mallaha (Eynan). In *Natufian Foragers in the Levant*. *Terminal Pleistocene Social Changes in Western Asia* (Eds. Bar-Yosef, O & Valla, F.R.) 312-318 (International Monographs in Prehistory, Ann Harbor, 2013).
11. Rosen, A.M. Natufian plant exploitation: Managing risk and stability in an environment of change. *Eurasian Prehistory* **7**, 117–131 (2010).
12. Valla, F.R. et al. Eynan (‘Ain Mallaha). In *Quaternary of the Levant* (Eds. Enzel, Y. & Bar-Yosef, O.) 291-294 (Cambridge University Press, Cambridge, 2017).
13. Perrot, J. Beisamoun. *Isr. Explor. J.* 16 (4), 271–272 (1966).
14. Valla, F.R., et al. Les fouilles de ‘Ain Mallaha (Eynan) de 2003 à 2005: Quatrième rapport préliminaire. *J. Isr. Preh. Soc.* **37**, 135–379 (2007).
15. Perrot, J. Premiers villages de Syrie et de Palestine. *C. R. Seanc. Acad. Sci.* **112**(2), 161–177 (1968).
16. Edwards, P.C. Problems of recognising earliest sedentism: The Natufian example. J*. Mediterr. Archaeol.* **2,** 5-48 (1989).
17. Valla, F.R. & Khalaily, H. The first sedentary peoples in Israel: Mallaha (Eynan) 1996. *Bull. Cent. Rech. Fr. Jérus.* **1**, 72–82 (1997).
18. Belfer-Cohen, A. & Bar-Yosef, O. Early Sedentism in the Near East: A Bumpy Ride to Village Life. In *Life in Neolithic Farming Communities: Social Organization, Identity, and Differentiation,* (Ed. Kuijt, I.) 19-38 (Kluwer Academic Publishers, United States of America, 2002).
19. Weissbrod, L. et al. Origins of house mice in ecological niches created by settled hunter-gatherers in the Levant 15,000 y ago. *Proc. Natl. Acad. Sci. USA* **114**(16), 4099-4104 (2017).
20. Valla, F.R. & Bocquentin, F. Les maisons, les vivants, les morts: le cas de Mallaha (Eynan), Israël. In *Houses for the Living and a Place for the Dead* (Eds. Balkan, N., Molist, M. & Stordeur, D.) 541-542 (ICAANE, Madrid, 2008).
21. Valla, F.R., Khalaily, H., Samuelian, N. & Bocquentin, F. What happened in the Final Natufian? *J. Isr. Preh. Soc.* ***40****,* 131–148 (2010).
22. Bocquentin, F. A final Natufian population: health and burial status at Eynan-Mallaha. In *Faces from the past: diachronic patterns in the biology of human populations from the Eastern Mediterranean. Papers in honour of Patricia Smith* (Eds. Faerman, M., Horwitz, L.K., Khana, T. & Zilberman, U.) 66-81 (Archaeopress, Oxford, 2007).
23. Ibáñez, J.J. et al. Rapport Qarassa 2010. Mission syro-Française de la Léja. Travaux de l'equipe espagnole (Unpublished report, Damascus, 2010a).
24. Braemer, F. et al. Long-term management of water in the central levant: the hawran case (Syria). *World Archaeol.* **41** (1), 36-57 (2009).
25. Braemer, F., Ibanez, J.J. & Shaarani, W. Qarassa (Mohafazat de Suweida): campagne 2009. *Chron. Archeol. Syr.* **5**, 31-42 (2011).
26. Ibáñez, J.J. et al. Rapport Qarassa 2009. Mission syro-française de la léja, travaux de l’equipe 358 espagnole (Unpublished report, Damascus, 2009).
27. Ibáñez, J.J. et al. The early PPNB levels of tell Qarassa North (Sweida, southern Syria). *Antiquity* **84**, 325 (2010b).
28. Arranz-Otaegui, A. et al. Landscape transformations at the dawn of agriculture in southern Syria (10.7–9.9 ka cal. BP): Plant-specific responses to the impact of human activities and climate change. *Quat. Sci. Rev.* **158**, 145-163 (2017).
29. Chikahli, M. & Amri, A. Jabal el- Arab: a mediterranean island. Dryland Agrobio 3, 8 (2008).
30. Traboulsi, M. Les précipitations dans les marges arides du Proche-Orient: l'exemple du bassin versant du Yarmouk. *Hannon Rev. Géogr. Libanaise* **26**, 7-39 (2013).
31. Arranz-Otaegui, A., Colledge, S., Ibáñez, J.J. & Zapata, L. Crop husbandry activities and wild plant gathering, use and consumption at the EPPNB Tell Qarassa North (south Syria). *Veg. Hist. Archaeobot.* **25** (6), 629-645 (2016a).
32. Arranz-Otaegui, A., Colledge, S., Zapata, L., Teira-Mayolini, L.C. & Ibáñez, J.J. Regional diversity on the timing for the initial appearance of cereal cultivation and domestication in southwest Asia. *Proc. Natl. Acad. Sci. USA* **113**(49), 14001-14006 (2016b).
33. Ibáñez, J.J., González-Urquijo, J.E. & Braemer, F. The human face and the origins of the Neolithic: the carved bone wand from Tell Qarassa North, Syria. *Antiquity* **88**, 81-94 (2014).
34. Balbo, A.L. et al. Squaring the circle. Social and environmental implications of pre-pottery neolithic building technology at tell Qarassa (south Syria). *PLoS One* **7**(7), e42109 (2012).
35. Santana, J. et al. Interpreting a ritual funerary area at the early Neolithic site of tell Qarassa North (south Syria, late 9th millennium BC). J*. Anthropol. Archaeol.* **37**, 112-127 (2015).
36. Santana, J., Velasco, J., Ibanez, J.J. & Braemer, F. Crania with mutilated facial skeletons: a new ritual treatment in an early Pre-Pottery Neolithic B cranial cache at Tell Qarassa North (South Syria). *Am. J. Phys. Anthropol.* **149**(2), 205-216 (2012).
37. Edwards, P.C. & Thorpe, S. Surface Lithic Finds at Kharaysin, Jordan. *Paléorient* **12**(2), 85-87 (1986).
38. Ibáñez, J.J. et al. 2019. Los primeros agricultores y ganaderos. Excavaciones en el yacimiento del Neolítico Precerámico A y B de Kharaysin (Zarqa, Jordania): campañas de 2015 y 2016. *Informes y trabajos* **17**, 103–23.
39. Moník, M. et al. Revealing early villages: pseudo-3D ERT geophysical survey at the pre-pottery Neolithic site of Kharaysin, Jordan. *Archaeol. Prospect.* **25**, 339–346 (2018).
40. Santana, J. et al., Transforming the ancestors: early evidence of fire-induced manipulation on human bones in the Near East from the Pre-Pottery Neolithic B of Kharaysin (Jordan). *Archaeol. Anthropol. Sci.* **12**, 112 (2020).
41. Ibáñez, J.J. et al. Flint ‘figurines’ from the Early Neolithic site of Kharaysin, Jordan. *Antiquity* **94**, 880-899 (2020).
42. Rollefson, G.O., Simmons, A.H. & Kafafi, Z.A.K. Neolithic Culture at 'Ain Ghazal. *J. Field Archaeol.* **19**, 443-70 (1992).
43. Rollefson, G.O., Kafafi, Z. A. K. & Kehrberg, I. The 1996 season at ʻAyn Ghazal. Preliminary report. *Annual of the Department of Antiquities of Jordan* **41**, 27-48 (1997).
44. Neef, R. PPNB settlements: vegetation and climate. A comparison between PPNB ‘ain ghazal and Basta. In *Central Settlements in Neolithic Jordan* (Eds. Bienert, H.D., Gebel, H.G.K., Neef, R.) 289-299 (ex Oriente, Berlin, 2004a).
45. Neef, R. Vegetation and plant husbandry In *Basta I: The Human Ecolog*y (Eds. Nissen, H.J., Muheisen, M. & Gebel, H.G.K.) 187-218 (ex Oriente, Berlin, 2004b).
46. Takahashi, K. & Arakawa, H. Climates of Southern and Western Asia. (Elsevier Scientific Publishing Company, Amsterdam, 1981).
47. Rollefson, G.O. & Köhler-Rollefson I. PPNC adaptations in the first half of the 6th millennium B.C. *Paléorient* **19 (**1), 33–42 (1993).
48. Rollefson, G.O. & Köhler-Rollefson, I. The collapse of Early Neolithic settlements in the southern Levant. In *People and Culture in Change: Proceedings of the Second Symposium on Upper Palaeolithic, Mesolithic and Neolithic Populations of Europe and the Mediterranean Basin* (Ed. Hershkovitz, I.) 73-89 (BAR International Series 508, Oxford, 1989).
49. Rollefson, G.O. Ritual and social structure at Neolithic ‘Ain Ghazal. In *Life in Neolithic Farming Communities: Social Organization, Identity, and Differentiation,* (Ed. Kuijt, I.) 165-190 (Kluwer Academic Publishers, United States of America, 2002).
50. Köhler-Rollesfon, I. Changes in goat exploitation at ‘Ain Ghazal between the Early and Late Neolithic: a metrical analysis. *Paléorient* **15**, 141–46 (1989).
51. von den Driesch, A. & Wodtke, U. The Fauna of ‘Ain Ghazal, a major PPN and early PN settlement in Central Jordan. In *The Prehistory of Jordan II. Perspectives from 1997* (Eds. Gebel, H.G.K., Kafafi, Z.A.K. & Rollefson, G.O.) 511-556 (ex Oriente, Berlin, 1997).
52. Makarewicz, C.A. Sequential δ13C and δ18O analyses of early Holocene bovid tooth enamel: Resolving vertical transhumance in Neolithic domesticated sheep and goats. *Palaeogeogr. Palaeoclimatol. Palaeoecol.* 485, 16-29 (2017).
53. Martin. L. & Edwards, Y. Diverse strategies: evaluating the appearance and spread of domestic caprines in the southern Levant. In *The Origins and Spread of Domesticated Animals in Southwest Asia and Europe* (Eds. Colledge, S., Conolly, J., Dobney, K., Manning, K., Shennan, S.) 49-82 (Left Coast Press, Walnut Creek, 2013).
54. Lechevallier, M. *Abou Gosh et Beisamoun: deux gisements du VIIe millénaire avant l’ère Chrétienne en Israël* (Associations Paléorient, Paris, 1978).
55. Bocquentin, F. et al. Renewed excavations at Beisamoun: investigating the 7th millennium cal. BC of the Southern Levant. *J. Israel Prehist. Soc.* 44, 5–100 (2014).
56. Bocquentin, F et al. Between Two Worlds: The PPNB-PPNC Transition in the Central Levant as Seen Through Discoveries at Beisamoun. In *The Mega-Project at Motza (Moza): The Neolithic and Later Occupations up to the 20th Century, New Studies in the Archaeology of Jerusalem and Its Region* (Eds. Khalaily, H., Re’em, A., Vardi, J., Milevski, I.) 163-199 (Israel Antiquity Authorities, Jerusalem).
57. Bocquentin, F., Barzilai, O., Khalaily, H. & Horwitz, L.K. The PPNB Site of Beisamoun (Hula Basin): Present and Past Research. In *The state of the stone: Terminologies, Continuities and Contexts in Near Eastern Lithics. Studies in early Near Eastern production, subsistence, and environment* (Eds. Healey, E., Campbell, S., Maeda, O.) 197-211 (ex Oriente, Berlin, 2011).
58. Borrell, F., Bocquentin, F., Gibaja, J. & Khalaily, H. Defining the Final PPNB/PPNC in the Southern Levant: insights from the chipped stone industries of Beisamoun. In *Near Eastern Lithic Technologies on the Move: Interactions and Contexts in Neolithic Traditions: 8th International Conference on PPN Chipped and Ground Stone Indistries of the Near East, Nicosia, November 23rd-27th 2016* (Eds. Astruc, L., McCartney, C., Briois, F. & Kassianidou, V.) 381-400 (Astrom Editions: Studies in Mediterranean Archaeology, Uppsala, 2019).
59. Rosenberg, D. *An Early Pottery Neolithic occurrence at Beisamoun, the Hula Valley, Northern Israel: the results of the 2007 salvage excavation* (BAR International Series 2095/Archaeopress, Oxford, 2010).
60. Wright, L.E. Identifying immigrants to Tikal, Guatemala: defining local variability in strontium isotope ratios of human tooth enamel. *J. Arch. Sci.* 32(4), 555-566 (2005).
61. Knudson, K.J. & Tung, T.A. Investigating regional mobility in the southern hinterland of the Wari Empire: biogeochemistry at the site of Beringa, Peru. *Am. J. Phys. Anthropol.* **145**(2), 299- 310 (2011).
62. Perry, M.A., Coleman, D. & Delhopital, N. Mobility and exile at 2nd century A.D. Khirbet edh-Dharih: Strontium isotope analysis of human migration in western Jordan. *Geoarchaeology* **23**, 528–549 (2008).
63. Sneh, A., Bartov, Y., Weissbrod, T. & Rosensaft, M. *Geological Map of Israel, 1:200,000* (*Isr. Geol. Surv.,* Jerusalem, 1998).
64. Hartman, G. & Richards, M. Mapping and defining sources of variability in bioavailable strontium isotope ratios in the Eastern Mediterranean. *Geochim. Cosmochim. Acta* **126**, 250–264 (2014).
65. Perry, M.A., Coleman, D., Dettman, D. & Al-Shiyab, A.H. An isotopic perspective on the transport of Byzantine mining camp laborers into southwestern Jordan. *Am. J. Phys. Anthropol.* **140** (3), 429–441 (2009).
66. Shewan, L. Natufian settlement systems and adaptive strategies: The issue of sedentism and the potential of strontium isotope analysis. In *The last hunter-gatherers in the Near East* (Ed. Delage, C.) 55–94 (Archaeopress/BAR international series 1320, Oxford, 2004).
67. Stein, M. et al. Sr-isotopic, chemical, and sedimentological evidence for the evolution of Lake Lisan and the Dead Sea. *Geochim. Cosmochim. Acta* **61**, 3975–3992 (1997).
68. Spiro, B., Ashkenazi, S., Starinsky, A., & Katz, A. Strontium isotopes in Melanopsis sp. as indicators of variation in hydrology and climate in the Upper Jordan Valley during the Early–Middle Pleistocene, and wider implications. *J. Hum. Evol.* **60**(4), 407-416 (2011).
69. Gregoricka, L.A., Ullinger, J. &Sheridan, S.G. Status, kinship, and place of burial at Early Bronze Age Bab adh-Dhra': A biogeochemical comparison of charnel house human remains. *Am. J. Phys. Anthropol.* **171**, 319–335 (2020).
70. Sharkov, E.V. et al. Geochronology of late Cenozoic basalts in western Syria. *Petrology* **2**, 385–394 (1994).
71. Dawod, S., Al-Mishwat, A. & Al Abdalla, A. Pliocene volcanic activity of the Harrat Ash-Sham, South of Syria: geochemistry and petrogenesis. *Iran. J. Earth. Sci.* **9**(1), 31-38 (2017).
72. Ponicarov, V.P. et al. *The geological map of Syria, scale 1/1,000,000* (Ministry of Industry of Syria, Damascus, 1966).
73. Krienitz, M.-S. et al. Tectonic events, continental intraplate volcanism, and mantle plume activity in northern Arabia: constraints from geochemistry and Ar–Ar dating of Syrian lavas. *Geochem. Geophys.* **10**, Q04008 (2009).
74. Bender, F. Geology of Jordan (Gebr. Borntraeger, Berlin, 1974).
75. Bender, F. Geology of the Arabian Peninsula: Jordan (US Government Printing Office, Washington D.C., 1975).
76. Henton, E. et al. Epipalaeolithic and Neolithic gazelle hunting in the Badia of north-east Jordan. Reconstruction of seasonal movements of herds by stable isotope and dental microwear analyses. *Levant* **50**, 127-172 (2018a).
77. Henton, E. et al. The seasonal mobility of prehistoric gazelle herds in the Azraq Basin, Jordan: modelling alternative strategies using stable isotopes. *Environ. Archaeol.* **23**(2), 187-199 (2018b).
78. Gregoricka, L.A. & Sheridan, S.G. Continuity or conquest? A multi‐isotope approach to investigating identity in the Early Iron Age of the Southern Levant. *Am. J. Phys. Anthropol.* **162**(1), 73-89 (2017).
79. Makarewicz, C.A. More than meat: diversity in caprine harvesting strategies and the emergence of complex production systems during the Late Pre-Pottery Neolithic B. *Levant* **45**, 236-261. (2013).
80. Gat, J.R. & Dansgaard, W. Stable isotope survey of the fresh water occurrences in Israel and the Northern Jordan Rift Valley. *J. Hydrol.* **16**, 177-211 (1972).
81. McLaren, S.J., Leng, M.J., Knowles T. & Bradley, A.V. Evidence of past environmental conditions during the evolution of a calcretised Wadi System in Southern Jordan using stable isotopes. *Palaeogeogr. Palaeoclimatol. Palaeoecol.* **348**, 1-12 (2012).
82. Al Charideh, A.R. & Abou Zakhem, B. Distribution of tritium and stable isotopes in precipitation in Syria. *Hydrol. Sci. J.* **55**(5), 832-843 (2010).
83. Bajjali, W. Spatial variability of environmental isotope and chemical content of precipitation in Jordan and evidence of slight change in climate. *Appl. Water Sci.* **2**(4), 271-283 (2012).
84. Bajjali, W. & Abu-Jaber, N. Climatological signals of the paleogroundwater in Jordan. *J. Hydrol.* **243**(1–2), 133–147 (2001).
85. Schoeninger, M.J. & Moore, K. Bone stable isotope studies in archaeology. *J. World Prehist.* **6**(2), 247-296 (1992).
86. Shomer-Ilan, A., Nissenbaum, A. & Waisel, Y. Photosynthetic pathways and the ecological distribution of the chenopodiaceae in Israel. *Oecologia* **48**(2), 244-248 (1981).
87. Hartman, G. & Danin, A. Isotopic values of plants in relation to water availability in the Eastern Mediterranean region. *Oecologia* **162**(4), 837-852 (2010).
88. Yamori, W., Hikosaka, K. & Way, D.A. Temperature response of photosynthesis in C3, C4, and CAM plants: temperature acclimation and temperature adaptation. *Photosynth. Res.* **119**(1-2), 101-117 (2014).
89. Dupras, T.L. & Tocheri, M. W. Reconstructing infant weaning histories at Roman period Kellis, Egypt using stable isotope analysis of dentition. *Am. J. Phys. Anthropol.* **134**(1), 63-74 (2007).
90. Krueger, H.W. & Sullivan, C.H. Models for carbon isotope fractionation between diet and bone. In *Stable Isotopes in Nutrition. American Chemical Society Symposium Series 258* (Eds. Turnlund, J.E. & Johnson, P.E.) 205**-**222 (American Chemical Society, Washington D.C., 1984).
91. Tieszen, L.L. & Fagre, T. Effect of diet quality and composition on the isotopic composition of respiratory CO2, bone collagen, bioapatite and soft tissues. In *Prehistoric Human Bone: Archaeology at the Molecular Level* (Eds. Lambert, J.B. & Grupe, G.) 121-155 (Springer-Verlag, Berlin/Heidelberg,1993).
